# The human gut microbiota contributes to type-2 diabetes non-resolution 5-years after Roux-en-Y gastric bypass

**DOI:** 10.1080/19490976.2022.2050635

**Published:** 2022-04-18

**Authors:** Jean Debédat, Tiphaine Le Roy, Lise Voland, Eugeni Belda, Rohia Alili, Solia Adriouch, Pierre Bel Lassen, Kazuyuki Kasahara, Evan Hutchison, Laurent Genser, Licia Torres, Camille Gamblin, Christine Rouault, Jean-Daniel Zucker, Nathalie Kapel, Christine Poitou, Geneviève Marcelin, Federico E. Rey, Judith Aron-Wisnewsky, Karine Clément

**Affiliations:** a Nutrition and obesities; systemic approaches (NutriOmics), Sorbonne Université, INSERM, Paris France; b IntegrativePhenomics, Paris, France; c Assistance Publique Hôpitaux de Paris, Pitié-Salpêtrière Hospital, Nutrition Department, France; d Department of Bacteriology, University of Wisconsin-Madison, Madison, Wisconsin, USA; e Visceral Surgery Department, Assistance Publique Hôpitaux de Paris, Pitié-Salpêtrière Hospital, France; f Unité de Modélisation Mathématique et Informatique des Systèmes Complexes, UMMISCO, Sorbonne Universités, Institut de Recherche pour le Développement (IRD), France; g Functional Coprology Department, Assistance Publique Hôpitaux de Paris, Pitié-Salpêtrière Hospital, France

**Keywords:** Microbiota, bariatric surgery, diabetes remission, bacteroides, fecal matter transplantation, relapse, type-2 diabetes, clustering, roux-en-Y gastric bypass, obesity

## Abstract

Roux-en-Y gastric bypass (RYGB) is efficient at inducing drastic albeit variable weight loss and type-2 diabetes (T2D) improvements in patients with severe obesity and T2D. We hypothesized a causal implication of the gut microbiota (GM) in these metabolic benefits, as RYGB is known to deeply impact its composition. In a cohort of 100 patients with baseline T2D who underwent RYGB and were followed for 5-years, we used a hierarchical clustering approach to stratify subjects based on the severity of their T2D (Severe vs Mild) throughout the follow-up. We identified via nanopore-based GM sequencing that the more severe cases of unresolved T2D were associated with a major increase of the class Bacteroidia, including 12 species comprising *Phocaeicola dorei, Bacteroides fragilis*, and *Bacteroides caecimuris*. A key observation is that patients who underwent major metabolic improvements do not harbor this enrichment in Bacteroidia, as those who presented mild cases of T2D at all times. In a separate group of 36 patients with similar baseline clinical characteristics and preoperative GM sequencing, we showed that this increase in Bacteroidia was already present at baseline in the most severe cases of T2D. To explore the causal relationship linking this enrichment in Bacteroidia and metabolic alterations, we selected 13 patients across T2D severity clusters at 5-years and performed fecal matter transplants in mice. Our results show that 14 weeks after the transplantations, mice colonized with the GM of Severe donors have impaired glucose tolerance and insulin sensitivity as compared to Mild-recipients, all in the absence of any difference in body weight and composition. GM sequencing of the recipient animals revealed that the hallmark T2D-severity associated bacterial features were transferred and were associated with the animals’ metabolic alterations. Therefore, our results further establish the GM as a key contributor to long-term glucose metabolism improvements (or lack thereof) after RYGB.

## Introduction

Despite decades of public efforts, the prevalence of obesity and related comorbidities (including type 2 diabetes (T2D)) are still rising worldwide.^1^ Obesity is a complex and multifactorial disease, intertwining host biology, genetics, and environmental factors.^2^ Recently, the gut microbiota (GM) has been described as a key contributor to both obesity and T2D, as alterations of its composition, richness, and functionality are associated with obesity^3^ and metabolic alterations, such as insulin resistance, low-grade systemic inflammation, and adiposity.^4–6^ For the most severe cases of obesity (BMI ≥40 kg/m^2^ or ≥35 kg/m^2^ when associated with comorbidities), bariatric surgery remains the only intervention able to induce major and long-lasting weight loss^7^ alongside significant improvements of all obesity-associated comorbidities.^8^ T2D remission (DR), defined as the quasi-normalization of HbA1C and fasting blood glucose values in the absence of glucose-lowering agents,^9^ concerns 50% to 60% of patients after bariatric surgery, although the prevalence of DR lessens with time.^10,11^ Several clinical parameters are associated with these differences in clinical outcomes, such as T2D duration, post-operative weight regain, and T2D severity, as proxied by the required number of glucose-lowering drugs.^10^ These factors are included in predictive scores able to estimate patients’ potential of entering in DR after their bariatric intervention.^11,12^ Nevertheless, the accuracy of these scores is low for a non-negligible fraction of patients,^12^ which suggests the involvement of other parameters in the observed metabolic improvements.

Amongst the numerous mechanisms proposed as participating in post-bariatric surgery metabolic improvements,^13^ studies reported deep and long-lasting modulations of the composition and functionality of the GM, which are associated with weight reduction and metabolic improvements.^6,14–16^ Furthermore, low-powered case-control studies provided associations between GM profiles and T2D remission.^17–19^ However, they did not substantiate the causal participation of the GM in the observed metabolic benefits.

To this end, the transfer of complex microbial communities from healthy or diseased donors to recipient animals via fecal matters transplants (FMT) remains the gold standard.^20^ After bariatric surgery, two human-to-mice FMT studies with obese non-T2D donors showed a partial transfer of the host adiposity^16^ and weight loss amplitude,^21^ and two rodents-to-mice studies presented either a transfer of the host adiposity^22^ or moderate and discordant metabolic phenotypes.^23^ Indeed, Arora *et al*., found that post-RYGB ileal content altered the metabolic phenotype of animals upon FMT, whereas cecal contents improved their metabolic conditions.^23^ Therefore, the involvement of the GM in post-operative T2D improvements remains to be explored, as well as its implication in the severity of the persisting T2D in patients who underwent different trajectories of metabolic improvements after their bariatric intervention.

By combining human and preclinical investigations, we aimed to decipher the participation of the GM in the long-term improvement of T2D after bariatric surgery, as well as its implication in the severity of the persisting cases of T2D. In this direction, we compared GM profiles of patients according to the magnitude of T2D improvement and performed human-to-mice FMT to substantiate the causal involvement of the GM in the post-operative metabolic changes.

## Results

### Categorizing T2D severity and metabolic improvements after RYGB

We included n = 100 patients from our bariatric surgery cohort with baseline T2D who underwent Roux-en-Y gastric bypass (RYGB), and for whom we had access to clinical data before, 1, and 5-years after their intervention (Fig. S1). At 5-years (mean follow-up: 5.19 ± 0.8 years), 49% (n = 49) of the patients were in DR (HbA1C < 6.5% and fasting blood glucose < 7 mmol/L without any glucose-lowering medication^9^). Although this definition of DR enables patients’ classification based on their metabolic benefits after bariatric surgery, it does not account for the baseline severity of T2D and thus for the amplitude of metabolic improvements, nor for the severity of unresolved T2D. Indeed, the metabolic phenotypes of patients with persisting T2D (i.e., patients in non-DR) were highly variable, ranging from HbA1C/fasting blood glucose close to DR’s targets in the absence of glucose-lowering drug, to values well above the targets while requiring several glucose-lowering medications.

**Figure 1.**
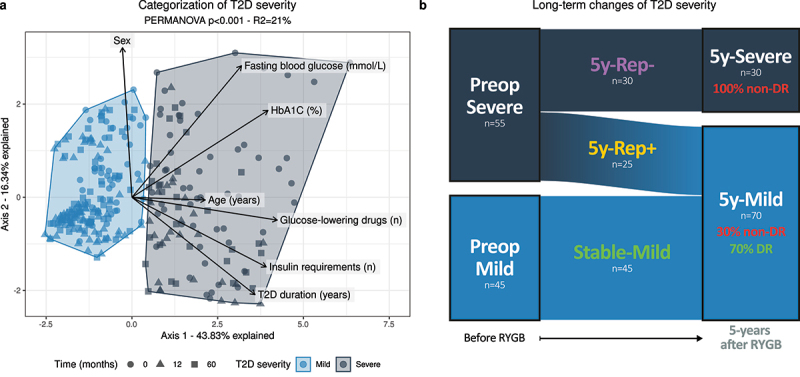
**Categorization and long-term evolution of T2D severity after RYGB (n = 100)**. (a) Patients’ PCA projection according to their T2D severity at all time points. ● represent patients’ baseline position, while ▲ and ■ indicate their position at 1- and 5-years post-RYGB, respectively. The arrows represent the intensity and the directionally of each variable’s contribution to the projection. (b) Long-term trajectories of T2D severity, and association with long-term T2D remission, which was defined according to the ADA’s criteria. 5y-, 5-years; DR, T2D remission; Rep+, good responders; Rep-, poor responders; RYGB, Roux-en-Y gastric bypass; T2D, type-2 diabetes.

This observed variability of metabolic phenotype prompted us to more accurately reclassify all 100 patients based on their metabolic phenotype at all time points (baseline, 1-year, and 5-years) before and after RYGB, and to define trajectories of metabolic improvements. To this end, we built clusters of T2D severity based on 7 clinical variables used as predictors for post-surgery metabolic improvements:^12,24^ HbA1C, fasting blood glucose, T2D duration, age, sex, number of glucose-lowering medications, and insulin requirements. These variables were integrated using principal component analysis (PCA), and clusters of T2D severity were defined by hierarchical clustering (Figure 1a). These clusters will be designated hereafter as “Mild” or “Severe”, and prefixes indicating the time-point of interest will be used (either “Preop”, “1y”, and “5y” for preoperative, 1 and 5-years, respectively).

The first component of the PCA translates the degree of T2D severity, as all variables except patients’ sex pointed toward the same direction (Figure 1a and S2). Standardized odd-ratios and 95% confidence intervals showed that HbA1C (OR 35.4, 95% CI [9.0–191.6] before and OR = 22.0, 95% CI [7.46–93.7] 5-years after RYGB), T2D duration (OR 12.8, 95% CI [4.8–46.1] before and OR 25.6, 95% CI [8.5–118.6] 5-years after RYGB) and the number of glucose-lowering drugs duration (OR 17.5, 95% CI [6.9–59.2] before and OR 20.1, 95% CI [7.3–78.7] 5-years after RYGB) were the most important variables defining T2D severity throughout the follow-up (Table S1). Of note, as all patients requiring insulin were in the Severe cluster both at baseline and 5-years after the intervention, their respective OR could not be computed due to the perfect separation. All 7 variables were compared across T2D severity clusters, and all (except sex) comparisons underlined the accurate classification of patients according to their metabolic phenotype (Fig. S3).

During the follow-up, the prevalence of patients in the Severe cluster decreased from 55% at baseline to 30% at 5-years, confirming an overall decrease in T2D severity after RYGB (Figure 1b). Most metabolic improvements occurred within the first year, and remained relatively stable afterward, as only 8% of patients changed clusters between years 1 and 5 (Fig. S4). Three trajectories of long-term T2D improvements were defined: good responders (“**5y-Rep+**”, Severe at baseline and Mild at 5-years), poor responders (“**5y-Rep-**”, Severe at all times), and finally metabolically “stable” patients (“**Stable-Mild**”, Mild at all times – Figure 1b).

Comparisons of baseline and 5-years clinical characteristics according to their trajectories revealed that all patients improved after the RYGB intervention, although with varying magnitude. At baseline, **5y-Rep+** and **5y-Rep-** patients (who were all in the Severe cluster) only differed on age, T2D duration, and insulin requirements (Table 1). However, **5y-Rep+** patients lost more weight and displayed striking metabolic improvements after RYGB, which led them to become clinically indistinguishable from **Stable-Mild** patients at 5-years, while **5y-Rep-** patients maintained an altered metabolic phenotype (Table 1). When only considering 5-years T2D severity clusters, patients in the **5y-Severe** cluster also presented more frequent and severe obesity-associated comorbidities (hypertension and dyslipidemia – Table S2). Importantly, no significant differences in body weight nor composition were found across trajectories or T2D severity clusters (Table 1).

**Table 1.** Clinical characteristics before and after RYGB across 5-years T2D severity trajectories. Variables are presented as mean ± SD or as n (%). Variance analyses were adjusted for age and sex, and within-classes were assessed via Dunn’s tests. #: binomial models were adjusted for age and sex. P-values were further corrected using the Benjamini-Hochberg method. CDR, complete type-2 diabetes remission; PDR, partial type-2 diabetes remission; non-DR, non-type-2 diabetes remission; Rep+, good responders; Rep-, poor responders

Variables	n	Stable-Mild (n = 45)	5y-Rep+ (n = 25)	5y-Rep- (n = 30)	Anova	Stable-Mild *vs* 5y-Rep+	Stable-Mild *vs* 5y-Rep-	5y-Rep+ *vs* 5y-Rep-
**Before RYGB**								
Fasting blood glucose (mmol/L)	100	6.68 ± 1.0	9.42 ± 2.9	8.63 ± 3.3	**<0.001**	**<0.001**	**0.006**	0.639
HbA1C (%)	100	6.67 ± 0.6	8.14 ± 1.4	8.76 ± 2.2	**<0.001**	**<0.001**	**<0.001**	0.253
Age (years)	100	46.45 ± 10.3	44.99 ± 11.8	54.52 ± 8.5	**<0.001**	0.877	**<0.001**	**0.003**
T2D duration (years)	100	2.82 ± 2.9	5.42 ± 3.4	15.62 ± 6.2	**<0.001**	**0.004**	**<0.001**	**<0.001**
Glucose-lowering drugs (n)	100	0 = 15 (33%) 1 = 21 (47%) ≥2 = 9 (20%)	0 = 1 (4%) 1 = 2 (8%) ≥2 = 22 (88%)	0 = 0 (0%) 1 = 2 (7%) ≥2 = 28 (93%)	**<0.001**	**0.011**	**<0.001**	0.305
Metformin usage #	100	no = 16 (36%) yes = 29 (64%)	no = 4 (16%) yes = 21 (84%)	no = 9 (30%) yes = 21 (70%)	0.253	0.239	0.654	0.309
Insulin usage #	100	no = 45 (100%) yes = 0 (0%)	no = 16 (64%) yes = 9 (36%)	no = 4 (13%) yes = 26 (87%)	**<0.001**	**<0.001**	**<0.001**	**<0.001**
Sex #	100	F = 33 (73% M = 12 (27%)	F = 21 (84%) M = 4 (16%)	F = 27 (90%) M = 3 (10%)	0.238	0.547	0.103	0.639
Body weight (kg)	100	127.36 ± 26.0	130.33 ± 22.7	119.86 ± 19.2	0.724	0.632	0.807	0.432
BMI (kg/m^2^)	100	47.11 ± 8.5	48.58 ± 9.9	45.74 ± 7.8	0.772	0.632	0.599	0.708
Fat mass (% bw)	96	47.64 ± 5.9	48.82 ± 4.7	47.05 ± 4.7	0.263	0.969	0.227	0.150
Lean mass (% bw)	96	50.08 ± 5.7	48.95 ± 4.6	50.68 ± 4.5	0.253	0.969	0.222	0.150
Dyslipidaemia #	100	no = 21 (47%)yes = 24 (53%)	no = 8 (32%)yes = 17 (68%)	no = 5 (17%)yes = 25 (83%)	**0.037**	0.513	**0.010**	0.282
Lipid-lowering drugs (n)	100	0 = 34 (76%)1 = 10 (22%)≥2 = 1 (2%)	0 = 12 (48%)1 = 13 (52%)≥2 = 0 (0%)	0 = 7 (23%)1 = 22 (73%)≥2 = 1 (3%)	**<0.001**	0.077	**<0.001**	0.124
Hypertension #	100	no = 19 (42%)yes = 26 (58%)	no = 7 (28%)yes = 18 (72%)	no = 3 (10%)yes = 27 (90%)	**0.012**	0.513	**0.003**	0.157
Anti-hypertensive drugs (n)	100	0 = 22 (49%)1 = 8 (18%)≥2 = 15 (33%)	0 = 8 (32%)1 = 5 (20%)≥2 = 12 (48%)	0 = 3 (10%)1 = 10 (33%)≥2 = 17 (57%)	**0.002**	0.426	**<0.001**	0.098
**5-years after RYGB**								
T2D remission status #	100	CDR = 18 (40%) PDR = 16 (36%) non-DR = 11 (24%)	CDR = 6 (24%) PDR = 9 (36%) non-DR = 10 (40%)	CDR = 0 (0%)PDR = 0 (0%) non-DR = 30 (100%)	**<0.001**	0.413	**<0.001**	**0.004**
Fasting blood glucose (mmol/L)	100	5.41 ± 1.1	5.76 ± 1.1	7.39 ± 1.9	**<0.001**	0.239	**<0.001**	**0.004**
HbA1C (%)	100	6.03 ± 0.5	6.13 ± 0.6	7.58 ± 1.0	**<0.001**	0.548	**<0.001**	**<0.001**
Age (years)	100	51.76 ± 10.1	50.73 ± 12.2	60.41 ± 8.6	**<0.001**	0.969	**<0.001**	**0.003**
T2D duration (years)	100	8.13 ± 2.9	11.16 ± 3.4	21.51 ± 6.3	**<0.001**	**0.001**	**<0.001**	**<0.001**
Glucose-lowering drugs (n)	100	0 = 40 (89%) 1 = 5 (11%) ≥2 = 0 (0%)	0 = 22 (88%) 1 = 2 (8%) ≥2 = 1 (4%)	0 = 3 (10%) 1 = 8 (27%) ≥2 = 19 (63%)	**<0.001**	0.969	**<0.001**	**<0.001**
Metformin usage #	100	no = 41 (91%) yes = 4 (9%)	no = 22 (88%) yes = 3 (12%)	no = 10 (33%) yes = 20 (67%)	**<0.001**	0.832	**<0.001**	**<0.001**
Insulin usage #	100	no = 45 (100%) yes = 0 (0%)	no = 25 (100%) yes = 0 (0%)	no = 16 (53%) yes = 14 (47%)	**<0.001**	-	**<0.001**	**<0.001**
Body weight (kg)	100	99.59 ± 24.6	92.86 ± 20.9	88.37 ± 16.9	0.625	0.548	0.587	0.864
BMI (kg/m^2^)	100	36.77 ± 8.0	33.52 ± 6.2	33.58 ± 6.3	0.241	0.239	0.408	0.783
Fat mass (% bw)	76	42.60 ± 7.1	43.02 ± 5.6	42.82 ± 5.3	0.724	0.632	0.572	0.666
Lean mass (% bw)	76	54.08 ± 6.2	53.93 ± 5.3	54.60 ± 5.3	0.537	0.547	0.511	0.642
Dyslipidaemia #	100	no = 24 (53%) yes = 21 (47%)	no = 11 (44%) yes = 14 (56%)	no = 8 (27%) yes = 22 (73%)	0.100	0.632	**0.033**	0.282
Lipid-lowering drugs (n)	100	0 = 41 (91%) 1 = 3 (7%) ≥2 = 1 (2%)	0 = 21 (84%) 1 = 4 (16%) ≥2 = 0 (0%)	0 = 16 (53%) 1 = 14 (47%) ≥2 = 0 (0%)	**0.001**	0.596	**<0.001**	**0.035**
Hypertension #	92	no = 20 (47%) yes = 23 (53%)	no = 11 (46%) yes = 13 (54%)	no = 1 (4%) yes = 24 (96%)	**<0.001**	0.969	**<0.001**	**0.001**
Anti-hypertensive drugs (n)	100	0 = 23 (51%) 1 = 11 (24%) ≥2 = 11 (24%)	0 = 15 (60%) 1 = 6 (24%) ≥2 = 4 (16%)	0 = 6 (20%) 1 = 16 (53%) ≥2 = 8 (27%)	**0.007**	0.632	**0.010**	**0.006**
1-year weight loss (% t0)	100	−25.06 ± 7.5	−30.21 ± 5.8	−25.97 ± 7.9	**0.048**	**0.036**	0.491	0.166
1-5-years weight evolution (% 1y)	100	4.52 ± 11.4	1.91 ± 8.2	0.09 ± 9.4	0.625	0.547	0.807	0.732
5-years weight loss (% t0)	100	−21.48 ± 12.7	−28.99 ± 7.2	−26.19 ± 8.2	**0.038**	**0.043**	0.488	0.312

Overall, our clustering approach differed from the definition of DR^9^ for n = 21/51 (41%) non-DR patients who were classified within the **5y-Mild** cluster (Table S3). While at baseline, these patients were clinically similar to patients in DR, they improved less after RYGB (Table S3). All 21 patients were close to DR’s targets, and 38% (n = 8/21) required minimal medications (mostly metformin), while 90% (n = 27/30) of patients within the **5y-Severe**/non-DR were well above DR’s targets despite taking glucose-lowering drugs (including insulin for 52% of them – Table S3). Therefore, our clustering approach allowed us to accurately identify patients’ trajectories translating the extent of their glucose-metabolism improvements.

### T2D severity is associated with specific gut microbiome alterations pre- and post-RYGB

Nanopore-based GM sequencings were performed using the MinION (ONT) on fecal samples collected 5-years post-RYGB for our 100 patients with long-term follow-up, as well as on 36 samples of an independent group of patients with identical baseline characteristics (referred to as the “baseline cohort”, see Table S4 and Fig S1) for whom we had access to pre-RYGB fecal samples. This high-throughput nanopore-based technology offers the possibility to sequence long DNA fragments without the need for pre-sequencing PCR amplification, as we recently published.^25^

**Figure 2.**
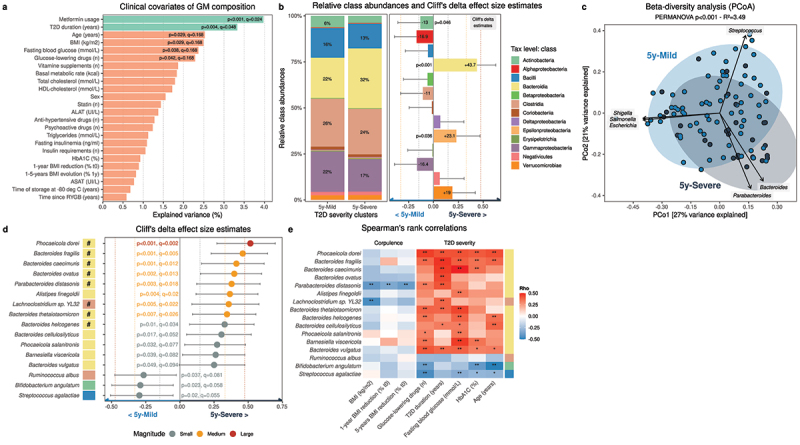
**T2D severity after RYGB is associated with specific GM signatures (n = 99)**. (a) Proportion of explained GM variation (dbRDA based on genus-level Bray-Curtis dissimilarity matrix). (b) Left: Relative class abundances representing at least 0.1% of the total ecosystem (n = 13) across T2D severity clusters. Right: Cliff’s delta effect size estimates, 95% confidence intervals and Welch Two-Samples t-tests p-values across T2D severity clusters and taxonomic classes representing at least 0.1% of the total ecosystem (n = 13). (c) Genus-level community PCoA ordination of Bray-Curtis dissimilarities. Arrows represent the 6 genera contributing the most to the ordination (R^2^ > 0.5). (d) Cliff’s delta effect size estimates, 95% confidence intervals and Welch Two-Samples t-tests p-values across T2D severity clusters and bacterial species representing at least 0.1% of the total ecosystem (n = 95). #-noted differences remain significant when controlled for metformin intake and cluster transition. (e) Heatmap of Spearman’s rank correlations between the abundance of the 16 species presented in Figure D, and 5-years clinical variables (*: p < .05; **: q < 0.1). 5y, 5-years; ASAT, aspartate aminotransferase; ALAT, alanine aminotransferase; BMI, body mass index; HDL, high density lipoprotein; PCoA, Principal Coordinate Analysis; RYGB, Roux-en-Y gastric bypass; T2D, type-2 diabetes.

We first assessed on our 5-years cohort the contribution of clinical variables and medications to overall GM composition. Univariate distance-based redundancy analysis (dbRDA) with a Bray-Curtis dissimilarity matrix revealed that two variables significantly explained GM variation: metformin intake and T2D duration (Figure 2a), although only metformin intake explained a non-redundant fraction of GM variation (R^2^_adj_ = 2.78, p = .002). Of note, parameters associated with fecal DNA extraction and sequencing did not significantly affect GM composition (Fig. S5).

When comparing relative class distributions across 5-years T2D severity clusters using Cliff’s delta estimates (CDe), we found that Actinobacteria were slightly less abundant in **5y-Severe** patients, whereas both Epsilonproteobacteria and Bacteroidia were significantly increased, the latter being the most prevalent class in **5y-Severe** patients (Figure 2b). At the genus level, a Principal Coordinate Analysis (PCoA) on a β-diversity matrix revealed a significant interaction between 5-years T2D severity and GM composition (Figure 2c). *Bacteroides, Parabacteroides, Streptococcus, Shigella, Escherichia, and Salmonella* were the 6 genera contributing the most to patients’ separation (R^2^ > 0.5, p < .001), and the directions of both *Bacteroides*/*Parabacteroides*’s contributions confirmed their association with T2D severity (Figure 2c).

Amongst species representing at least 0.1% of the total ecosystem (n = 95), 13 bacteria were significantly more prevalent in **5y-Severe** patients, while 3 were more abundant in **5y-Mild** patients (Figure 2d). Most species (12/13) enriched in **5y-Severe** patients are members of the Bacteroidia class, with *Phocaeicola dorei* having the highest association (CDe = 0.52, q = 0.002), followed by *Bacteroides fragilis* (CDe = 0.46, q = 0.004) and *Bacteroides caecimuris* (CDe = 0.416, q = 0.01). Interestingly, these 16 species are associated with clinical parameters of T2D severity and corpulence, further suggesting their link with post-RYGB clinical metabolic traits (Figure 2e). Of note, only 1 species from the class Actinobacteria (out of 13 species of Actinobacteria representing >0.1% of the total ecosystem) was significantly different according to patients’ T2D severity clusters, while 0/2 was from the Epsilonproteobacteria class, suggesting marginal differences in these two classes.

To gain further understanding of how these shifts in GM composition associated with T2D severity may impact the functions of the microbiome, we applied an inference-based method to generate a matrix of genes (using the KEGG database) based on patients’ GM composition data. By correlating the abundance of the 13 species associated with T2D severity to the abundance of inferred genes, we noticed that the increase in Bacteroides have an important impact on metabolic capacities (Fig. S6A). To confirm that these metabolic differences were not compensated by other species, we computed CDe for each of these metabolic pathways across 5-years T2D severity clusters (Fig. S6B). Several metabolic pathways, such as carbon and carbon fixation, glycosaminoglycan, branched-chain amino-acids and methane metabolisms were increased in 5y-Severe patients (p < .05, q < 0.1), whereas metabolism of other amino-acids (cysteine and methionine, glycine, serine and threonine, glutathione) was increased in 5y-Mild patients (Fig. S6B).

As **5y-Rep+** patients were, at 5-years, clinically similar to **Stable-Mild** patients (Table 1), we wondered whether their GM might harbor a signature associated with metabolic improvements. Compositional analysis revealed extremely similar profiles (Fig. S7A-C), as neither a PCoA on a β-diversity matrix nor differences of relative class abundances (estimated by CDe) accounted for any significant difference between the two trajectories. At the species level, CDe allowed for the discovery of only two differentially abundant species: *Enterococcus faecalis* (enriched in **Stable-Mild** patients), and *Tannerella forsythia* (enriched in **5y-Rep+** patients). However, **5y-Rep+** patients tended to present insignificant yet slightly increased abundances of several of the species associated with 5-years T2D severity. Overall, these observations led us to hypothesize that the transition of T2D severity clusters (Severe to Mild, which translates their major metabolic improvements) is associated with a partial restoration of a “Mild” profile of the GM.

Subsequently, we projected the n = 36 patients from the independent baseline cohort on the PCA built on our 5-years cohort (see Methods and Fig. S8A) to identify their preoperative T2D severity cluster. By studying their GM, we found that the important increase in Bacteroidia was already present pre-RYGB in Severe patients (Fig. S8B). *Tannerella forsythia* was also enriched in Preop-Severe patients (Fig. S8C). Of note, we did not evidence any difference in the number of observed species nor diversity indexes according to 5-years T2D severity clusters (Fig. S9A-G), although most significantly increased after RYGB (Fig. S9H-N), as previously reported.^6^

We demonstrate an increase in the Bacteroidia class in patients with severe cases of T2D severity both before and 5-years after RYGB. Importantly, patients who were in the Severe cluster at baseline and transitioned toward the 5y-Mild cluster at 5-years present a similar GM profile to patients who were in the Mild cluster all along. Importantly, most bacterial associations with 5-years T2D severity remain significant when controlling comparisons for metformin intake and cluster transitions (# on Figure 2d).

### Fecal transfers in mice induced a transfer of the donors’ metabolic alterations

To explore causality linking post-RYGB GM composition and T2D severity, human to mice fecal matter transfers (FMT) were performed. Nine **5y-Severe** and 4 **5y-Mild** patients were selected based on their clinical profiles (Table 2). To avoid any potentially confounding issue due to sex and medications, we selected **5y-Severe** women classified in 3 homogeneous groups of glucose-lowering agents (metformin and insulin, insulin but not metformin, or only metformin – see Table 2). All **5y-Mild** donors were free of anti-T2D medication. Patients were recalled at the hospital for fresh stool samples collection, with which we confirmed that their GM profiles were representative of their cluster. Out of the 16 species presented Figure 2d, similar differences across 5-years T2D severity clusters were observable within the selected donors, except for *Alistipes finegoldii* and *Bacteroides vulgatus* (Fig. S10).

**Table 2.** Clinical characteristics of patients selected as donors for the fecal microbiota transfer experiment. 1y, 1-year; 5y, 5-years; BMI, body mass index; DR, type-2 diabetes remission; non-DR, non-type-2 diabetes remission; t0, baseline; T2D, type-2 diabetes

**Donor**	5-years T2D severity cluster	5-years DR status	Treatment group	Age (years)	Body weight (kg)	BMI (kg/m^2^)	1-year weight loss (% t0)	1-5-years weight change (% 1y)	T2D duration (years)	HbA1C (%)	Fasting blood glucose (mmol/L)	Glucose-lowering drugs (n)	Insulin requirements (n)	Metformin usage
**Mild-1**	**5y-Mild**	**DR**	**None**	47.7	79	30.9	−24.9	5.9	6.2	5.5	5.5	0	no	no
**Mild-2**	**5y-Mild**	**DR**	**None**	59.2	74.8	30	−34.1	5.5	10.2	6.1	4.8	0	no	no
**Mild-3**	**5y-Mild**	**DR**	**None**	65.5	74.5	29.5	−39	2.2	17.5	5.2	4.3	0	no	no
**Mild-4**	**5y-Mild**	**DR**	**None**	68.1	77.5	27.5	−33.6	3.2	11.8	5.9	5.6	0	no	no
**Severe-1**	**5y-Severe**	**non-DR**	**Metformin + Insulin**	54.8	80	34.6	−23.1	4.7	23.6	7.3	3.7	≥2	yes	yes
**Severe-2**	**5y-Severe**	**non-DR**	**Metformin + Insulin**	55.2	86	32.4	−40.6	14.1	12.3	7	8.3	≥2	yes	yes
**Severe-3**	**5y-Severe**	**non-DR**	**Metformin + Insulin**	59.1	94	37.7	−17.4	−8	18.9	6.6	6.6	≥2	yes	yes
**Severe-4**	**5y-Severe**	**non-DR**	**Insulin only**	67.5	115	40.7	−14.6	−7.3	30.2	6.9	7	≥2	yes	no
**Severe-5**	**5y-Severe**	**non-DR**	**Insulin only**	59	68	28.3	−28.4	−19.8	25.1	7.9	7.7	≥2	yes	no
**Severe-6**	**5y-Severe**	**non-DR**	**Insulin only**	63.8	85	30.1	−16.6	−18.5	11	10.2	8.9	≥2	yes	no
**Severe-7**	**5y-Severe**	**non-DR**	**Metformin only**	68.9	69	31.5	−35.3	1	28.5	6.7	8.2	1	no	yes
**Severe-8**	**5y-Severe**	**non-DR**	**Metformin only**	60.3	79	29	−34.8	5.8	25.1	7.6	8.8	1	no	yes
**Severe-9**	**5y-Severe**	**non-DR**	**Metformin only**	64.3	67	24.6	−28.6	−5.5	17.8	7.2	8.7	1	no	yes
**Welch Two-Samples t-tests p-values**				0.94	0.44	0.33	0.41	0.20	**0.02**	**0.003**	**0.034**	**0.002**	0.105	0.105

**Figure 3.**
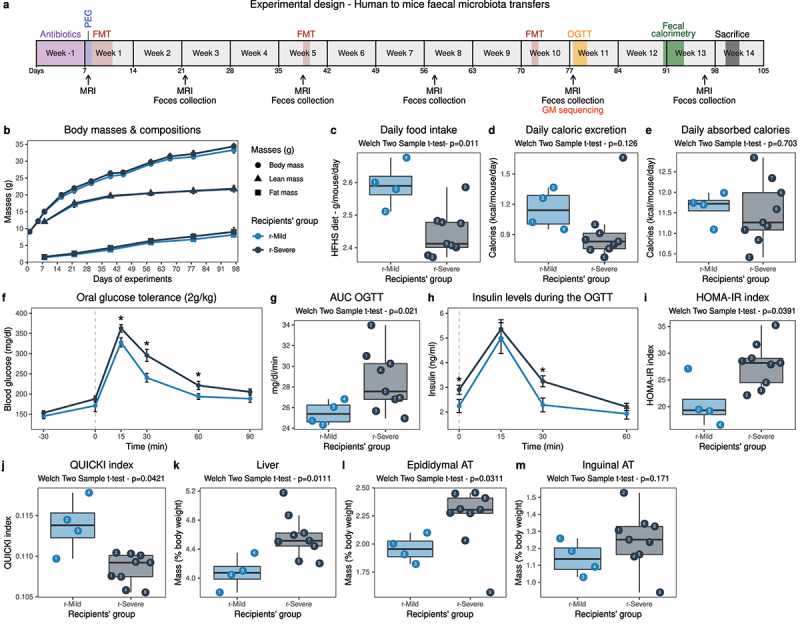
**Metabolic alterations were transferred upon fecal matters transfers (n = 52)**. (a) Experimental design. (b) Body (●), fat (■) and lean (▲) mass evolutions throughout the follow-up. (c) Daily food intake. (d) Daily fecal excretion of calories. (e) Daily caloric absorption. (f) Oral glucose tolerance test (dose: 2 g/kg) performed 10 weeks after the inoculation. (g) AUC of the glycemia measured during the OGTT. (h) Insulin levels quantified in plasma collected during the OGTT. (i) HOMA-IR index. (j) QUICKI index. (k-m) Liver, epididymal and inguinal adipose tissues masses. Numbers within the dots on boxplots represent the donor’s number according to Table 2. AT, adipose tissue; AUC, area under the curve; FMT, fecal microbiota transfer; HFHS, high-fat high-sucrose; OGTT, oral glucose tolerance test; MRI, magnetic resonance imaging; PEG, polyethylene glycol; r-, recipient.

FMT were performed during three consecutive days in juvenile conventional mice (n = 4 mice per donor, n = 52 in total) whose GM were depleted using broad-spectrum antibiotics and laxatives.^26^ Throughout the follow-up (Figure 3a), no difference in body weight nor composition were observed in the recipient animals (Figure 3b). Daily (Figure 3c) and cumulative food intakes (Fig. S11A-B) were higher in the **5y-Mild** recipients (“r-Mild”). As there was no difference in body weight, we hypothesized that the difference in food intake could be compensated by either decreased energy absorption or increased expenditure. We used bomb calorimetry to estimate the caloric content of the animals’ feces and found that the daily caloric excretion was higher in the r-Mild animals (Figure 3d) with a trend toward higher daily fecal output (Fig. S11C), which resulted in an overall decreased energy efficiency (Fig. S11D). Therefore, the amount of daily absorbed calories was similar between the two groups (Figure 3e).

To evaluate whether FMT induced alterations of the mouse’s metabolic phenotype, we performed an oral glucose tolerance test (OGTT) 10 weeks after the FMT. As compared to r-Mild animals, **5y-Severe** recipients (“r-Severe”) had a significantly impaired glucose tolerance, with significantly higher glycemia at the 15 minutes peak and slower clearance of blood glucose (Figure 3f-g). Insulin levels during the OGTT were significantly increased at fasting and at the 30 minutes time point in the r-Severe group, suggesting increased insulin resistance, in agreement with higher HOMA-IR and decreased QUICKI index values (Figure 3i-j).

When considering donors’ glucose-lowering agent subgroups (either metformin only, insulin without metformin, or metformin & insulin, see Table 2), both insulin-only and insulin+metformin recipient animals showed similar phenotypes (Fig. S12). Although these interpretations are limited by small sample sizes (n = 3 donors per treatment group), we found that recipients from metformin-only donors presented differences of intestinal absorption and glucose tolerance, closer to those of recipients from the non-treated (NT) patients. Conversely, they had the highest insulin secretion during the OGTT, and therefore showed the same alterations in insulin resistance/sensitivity indexes as other **5y-Severe** recipients (Fig. S12). Overall, these results suggest that the **5y-Severe** GM (regardless of the donors’ anti-T2D treatments) induced perturbations of glucose homeostasis in recipient animals. Finally, tissue harvesting revealed that r-Severe animals presented an increase in liver and visceral adipose tissue masses relative to their body weight (Figure 3k-m).

Importantly, this FMT study remains valid in the context of the DR/non-DR dichotomy, as all **5y-Mild** donors were in DR, and all **5y-Severe** donors were in non-DR (Table 2).

### Bacteroidia species linked with T2D-severity were transferred in recipient animals and associate with their altered metabolic phenotype

GM composition of recipient animals was determined 10 weeks after the initial inoculation, following the same protocols for DNA extraction and library sequencing used in patients.

**Figure 4.**
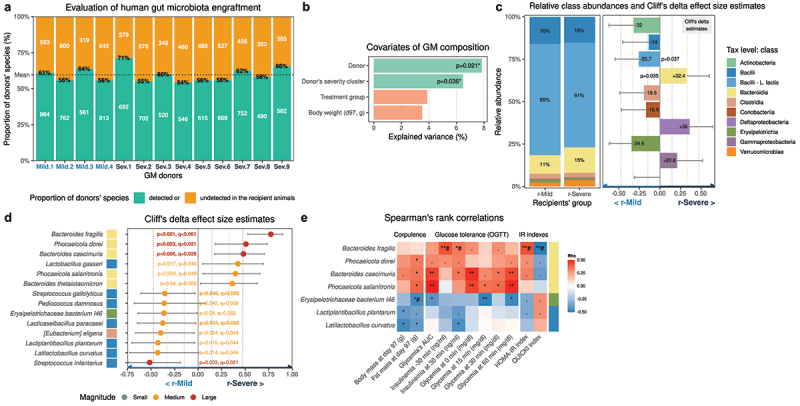
**The transfer of bacteria associated with T2D severity induces alterations of the recipients’ metabolic phenotype (n = 52)**. (a) Overall proportion of donor’s species detected in the recipient animals (proportion in green). (b) Proportion of GM variation (dbRDA based on genus-level Bray-Curtis matrix) explained by recipient animals’ groups and body weight. (c) Left: Relative class abundances across recipients’ clusters. Right: Cliff’s delta effect size estimates, 95% confidence intervals and Welch Two-Samples t-tests p-values across recipients’ clusters and taxonomic classes representing at least 0.1% of the total ecosystem (n = 10). (d) Cliff’s delta effect size estimates, 95% confidence intervals and Welch Two-Samples t-tests p-values between r-Mild and r-Severe animals across bacterial species representing at least 0.1% of the total ecosystem (n = 75 species in total). Bolded species remain significant when considering pseudoreplication. (e) Heatmap of Spearman’s rank correlations between the abundance of the 15 species presented in Figure C, and animals’ phenotypic variables (.: p < .1, *: p < .05; **: q < 0.1; FDR correction; #-noted relations remain significant (p < .05) when pseudoreplication is accounted for).

We first evaluated the long-term engraftment and persistence of the donors’ GM into the recipient animals. We found that a mean of 60% of the donor’s species was detected in their respective recipients, without any difference according to the donors’ clusters (p = .94 – Figure 4a). A major observation was that the donor and their respective T2D severity clusters are the variables explaining the highest proportion of variation within the recipients’ GM composition (Figure 4b), demonstrating that the FMT induced specific changes within the recipients’ GM (Figure 4b).

Evaluation of relative class abundances revealed a significant increase in Bacteroidia in r-Severe mice (Figure 4c). Amongst species representing at least 0.1% of the total GM (n = 75), 8 were more prevalent in r-Mild animals (CDe<0), and 6 in r-Severe animals (CDe>0 – Figure 4d). Five out of the 6 species increased in the r-Severe animals belonged to the class Bacteroidia and were also more prevalent in humans from the **5y-Severe** cluster (Figure 2d). The top three bacterial species, namely *Bacteroides fragilis, Phocaeicola dorei*, and *Bacteroides caecimuris*, were the top three species found to be associated with the **5y-Severe** cluster in humans (Figures 4d and 2d). When considering pseudoreplication, 5 species remained significantly different across the donors’ clusters: *Bacteroides fragilis, Phocaeicola dorei, Bacteroides caecimuris*, as well as two Bacilli: *Lacticaseibacillus paracasei* and *Streptococcus gallolyticus*.

Finally, we examined the relationships between the 15 bacterial species presented in Figure 4d and the phenotype of recipient animals. Amongst these species, several of which were increased in r-Severe animals and correlated with glucose tolerance variables (AUC, glycemia, and insulinemia all measured during the OGTT), with insulin-sensitivity indexes (HOMA-IR, QUICKI) and fat-mass (Figure 4e). Interestingly, most bacteria increased in r-Mild animals did not correlate with any glucose tolerance parameters, except for negative associations with *Erysipelotrichaceae bacterium I46, Lactiplantibacillus plantarum* and *Latilactobacillus curvatus* (Figure 4e).

Of note, 75% of the recipient animals’ GM is composed of species from the Bacilli class, mostly *Lactococcus lactis*, which represents by itself 60% of the total murine GM (Figure 4c). *Lactococcus lactis* has been described as a dietary contaminant of high-fat diets containing dairy byproducts,^27^ which was the case for our diet (see Methods).

## Discussion

By combining clinical and preclinical studies, we demonstrated that the severity of T2D is associated with an enrichment of the class Bacteroidia both before and after RYGB, which, we hypothesize, disappears upon major metabolic improvements. We moreover showed that human-to-mice FMT alters the metabolic phenotype of recipient animals through the transfer of species associated with T2D severity in humans. Therefore, it appears that the GM can be partly involved in post-RYGB metabolic improvements or lack thereof.

To overcome some limitations of the definition of DR^9^, including (i) lack of baseline T2D severity estimation and (ii) strict thresholds which cannot grasp the heterogeneity of persisting T2D, we used a hierarchical clustering approach to estimate the patient’s degree of glucose homeostasis deterioration before and after RYGB. A similar methodology demonstrated the ability to accurately classify T2D patients based on their risk of T2D-related complications.^28^ We confirmed the accuracy of the classification by showing that all the observed discrepancies with DR concerned patients in non-DR with very mild metabolic alterations with minimal glucose-lowering requirements. Most of all, this approach allowed us to identify trajectories of metabolic improvements, which are crucial in the search for mechanisms involved in bariatric surgery-induced metabolic improvements.

By analyzing patients’ GM composition 5-years after RYGB, we revealed a specific and metformin-independent enrichment of the Bacteroidia class associated with T2D severity and alterations of glucose homeostasis markers. A keystone observation is that the GM of good metabolic responders (**5y-Rep+**) is almost indistinguishable from that of **Stable-Mild** patients, both clinically and regarding GM composition. This thereby suggests that post-RYGB metabolic improvements (leading to cluster transition from Severe to Mild) are concomitant to a partial restoration of a “Mild” GM profile. We acknowledge that this finding requires external validations in cohorts with paired pre- and post-RYGB analysis of the GM. Nevertheless, GM changes in **5y-Rep+** patients, which also include an increase in the class Actinobacteria, are concordant to findings observed after T2D remission.^19^ Besides, the authors also observed no change in the abundance of Bacteroidia in non-DR patients, thus corroborating our observation in both baseline- and **5y-Severe** patients.

At the species level, the negative impact on the host of some of the bacteria associated with T2D severity was already proposed. Sun *et al*., showed that a metformin treatment in T2D individuals decreased the abundance of *Bacteroides fragilis*.^29^ In mice, they demonstrated that the supplementation of *Bacteroides fragilis* induced a complete blunt of metformin’s metabolic benefits.^29^ Intriguingly, they also observed that metformin reduced the abundance of several bacteria we found increased in **5y-Severe** patients. This raises questions regarding the long-term effect of metformin on the GM composition, as almost 70% of our patients in the **5y-Severe** cluster were taking metformin. Importantly, Sun *et al*., included newly diagnosed, untreated and barely overweight patients with mean HbA1C and fasting blood glucose values of 7.9% and 9.7 mmol/L, respectively.^29^ In contrast, our patients were severely obese, had a long history of T2D, and were sometimes receiving several other glucose-lowering drugs in addition to metformin. Therefore, we cannot exclude that the initial beneficial action of metformin on members of the Bacteroidia class might lessen with time in subjects with long-lasting histories of obesity- and T2D-associated GM dysbiosis. Moreover, the impact of metformin (and potentially other drugs^30^) on the GM may also differ between patients who underwent bariatric surgery and non-operated subjects, although this remains to be evaluated.

Reports on both *Bacteroides vulgatus* and *Phocaeicola dorei* are conflicting. *Bacteroides vulgatus* is associated with an altered metabolic profile, especially during severe obesity^6,31^ and is reduced upon a prebiotic treatment which moderately improves the host’s glucose homeostasis.^32^ Conversely, another study presented a positive impact of both *Bacteroides vulgatus* and *Phocaeicola dorei* on systemic inflammation and cardiovascular risk,^33^ which goes against another report presenting these two bacteria as pro-inflammatory.^34^ Intriguingly, *in-vivo* studies have demonstrated an inflammation-dampening effect (stimulation of IL-10 and reduction of IL-6) of *Bacteroides fragilis* which may participate to improvement of the animals’ glucose metabolism (as reviewed herein^35^), which seem to go against our observations detailed in the last paragraph. Furthermore, amongst the 3 species associated with a mild T2D severity, only *Streptococcus agalactiae* had been described in the context of metabolic diseases, as it was found in the subgingival microbiota of patients with impaired glucose homeostasis.^36^ *Tannerella forsythia*, which is significantly increased in **5y-Rep+** patients as compared to **Stable-Mild** patients, is also found in the subgingival microbiota of obese subjects^37^ and is decreased in patients with uncontrolled T2D.^38^ Overall, these conflicting results may arise from differences in population characteristics, disease states, medications, or bacterial strains, as two strains of the same species can induce distinct phenotypes.^34,39,40^ Besides, it suggests that both the gut and oral microbiota ought to be more extensively and causatively explored after bariatric surgery, especially in regard to metabolic improvements.

We also explored the metabolic consequences of such bacterial changes. Interestingly, our observations corroborate previous report made in the literature. For instance, studies have demonstrated that cysteine, methionine and glutathione levels (i) are associated with insulin resistance, (ii) can predict the risk of developing T2D,^41–43^ and (iii) are increased in mice submitted to a western NAFLD-inducing diet.^44^ As such, the decrease of these pathways in patients in the **5y-Severe** cluster suggest amino-acids metabolism impairments in persisting cases of T2D after bariatric surgery, and/or a semi-normalization of amino-acids metabolism after bariatric surgery in patients with metabolic improvements. On the other hand, the degradation of branched-chained amino-acids (BCAA) is increased in patients in the 5y-Severe cluster. Positive associations between BCAA and T2D/metabolic impairments have been observed multiple times throughout the years (as neatly reviewed here^45^). Besides, a very recent paper also demonstrated that the degradation of these BCAA could lead to the presence of branched-chained fatty acids, which negatively impact the host’s insulin sensitivity by impairing the mTORC1 signaling pathway.^46^ After bariatric surgery, BCAA possible participation to metabolic improvements has also been observed, as we reviewed herein.^13^ As such, one could argue that patients in the 5y-Severe cluster present more circulating BCAA/BCFA, which could in part explain their poorer metabolic health. This of course would need to be explored more deeply, for example by evaluating subjects’ feces metabolome. Changes in methane metabolism, glycosaminoglycan degradation and carbon fixation pathways in procaryotes were all found to be increased in T2D patients and in patients who later developed T2D in the study by Wang *et al*.,^47^ which concordant with our current findings.

In the second part of our study, we showed for the first time that hallmark GM alterations associated with T2D severity were partially transmittable in mice via FMT and were associated with an important alteration of the recipient animals’ metabolic phenotype. As previously mentioned, FMT studies in murine models of bariatric surgery presented limited effects on both adiposity and glucose homeostasis.^16,21,22,48^ Here, GM transfers were effective at inducing both GM and perturbations of glucose tolerance and insulin sensitivity. Furthermore, we found strong similarities between the donors and their recipient animals’ phenotypes, as well as between donors’ and recipient-specific bacterial abundances. These results confirm the partial involvement of the GM in T2D severity and persistence after RYGB.

Our model of conventional juvenile microbiota-depleted mice was proposed as an alternative to germ-free rodents.^26^ This model has shown satisfactory engraftment capabilities after human-to-mice FMT.^49–51^ Here, 60% of the detected species within the recipient animals were found in their respective donors, a level concordant with the literature and to the level of axenic animals.^52^ We chose to use multiple donors, which allowed for a relative diversity in terms of patients’ phenotype and medications, which are known to impact the GM.^30,53^ Moreover, we followed the recommendations proposed by Walter and colleagues^20^ to limit statistical biases due to pseudoreplication (artificial inflation of the number of experimental units). Overall, we believe that these methodological precautions strengthen our observations.

Nevertheless, we are aware of some limits. Although this study is, to date, the largest dedicated at studying GM composition in the context of long-term metabolic improvements post-RYGB, it suffers from relatively low power due to (i) difficulty to collect fecal samples in hospital settings, and to (ii) a relatively high attrition rate that we already discussed.^11^ Besides, most of our patients were women of Caucasian descent who all underwent RYGB. Therefore, our observations ought to be replicated on more diverse populations and different bariatric intervention types. Even though our clustering methodology proved efficient at classifying patients’ metabolic phenotypes both before and after bariatric surgery, we acknowledge that it may also result in false positive and/or false negatives within the assignments in other settings/populations. Further validations in large cohorts are required. Regarding GM analyses, the 2 time-points of ONT sequencing were not matched, limiting results interpretation at the individual level. Furthermore, ONT is discussed as an error-prone technique, although we recently demonstrated consistent results between ONT, Illumina, and SOLiD sequencing.^25^ We also acknowledge that the gene-inference methodology we use to study metabolic changes within patient’s GM has some limitations. May it reflect the functional “landscape” of the microbiome, it does not prove that these functional changes actually occur *in-situ*. Having data about circulating and fecal metabolites would be a way to address deeper the potential mechanisms linking the aforementioned featured associated with T2D severity and persistence of T2D after bariatric surgery. However, it was unfortunately not possible for us to perform this experiment in the current study. Furthermore, we did not explore dietary habits, socioeconomical status and lifestyle variables in the present cohort, as these data were not available for all of our patients and we observed a bias of response rates (a higher proportion of 5y-Mild patients answered the questionnaires). We acknowledge this as a limitation of our study, as these components may have an important impact on patient’s trajectories of metabolic improvements, their GM composition, and the abundance of the bacterial candidates we identified within this study. For the FMT experiment, the GM of women were transferred in male recipient animals. While not perfect, we chose this experimental design to ensure (i) a sufficient number of donors, as a large majority of patients in our cohort (and more generally in bariatric populations) are women, and (ii) to ensure the development of metabolic alterations via a high-fat-diet, toward which female mice are usually less suseptible.^54,55^ Even though sex is a very minimal contributor to subjects’ GM composition,^56,57^ we believe that our observations would benefit from confirmation with male donors or sex-matched recipient animals.

In conclusion, we herein report an accurate clustering method to grasp the magnitude of T2D improvements after RYGB, which allowed for the identification of a net enrichment in Bacteroidia in severe cases of T2D both before and 5-years after RYGB. Parts of the Bacteroidia fraction of the GM were transferred upon FMT and negatively impacted the glucose homeostasis of recipient mice, confirming a contribution of the GM in long-term T2D persistence and severity after RYGB. In addition, we observed that post-RYGB cluster transitions due to important metabolic improvements are associated with a strong decrease in Bacteroidia. This suggests that restoring a “healthier” GM profile (by eventually promoting other bacterial classes/species via dietary and/or lifestyle interventions,^58^ pre- or probiotics,^32,59^ drugs,^30,53^ or even FMT from healthy subjects^60^) may help improve the metabolic phenotype of patients both before and after bariatric surgery. There is now a need to examine the benefits of such interventions in pre- and post-bariatric settings.

## Materials and methods

### Study design and participants

We studied two subgroups of 136 patients in total, from our prospective bariatric surgery cohort “BARICAN” (Bariatric Surgery Cohort of Institute of Cardiometabolism and Nutrition) who had T2D at baseline, underwent RYGB, and for whom we had access to either stool samples collected during the 4-7-years clinical follow-up appointment for the first subgroup (n = 100) or to preoperative fecal samples for the second subgroup (n = 36 – Fig. S1).

### Study approval

As part of usual patient’s care, the BARICAN cohort is recruited and followed-up at the Pitié-Salpêtrière Hospital Nutrition Department (Paris, France), and is approved by CNIL (Commission Nationale de l’Informatique et des Libertés; No. 1222666) as well as the French Ministry of Research. Patients provided informed consent and are part of several studies registered on https://clinicaltrials.gov (P050318 Les Comités de Protéction des Personnes (CPP) approval: 24 November 2006, NCT01655017, NCT01454232). All patients met standard bariatric surgery indications^61^ and are monitored according to national and international guidelines. Patients were not involved in the design, conduct, reporting, or dissemination plans of this work.

### Clinical, bioclinical, and anthropometric variables

The following data were collected for all patients at every time point of follow-up, as previously described:^11^ anthropometric parameters, obesity-related comorbidities (including hypertension, T2D, dyslipidemia), the total list of treatments, T2D duration, bio-clinical data including blood tests (lipid panels, liver enzymes, and glucose-related parameters) as well as body composition measured by body DEXA scans (Hologic Discovery, West Bedford, MA). Fold changes of clinical variables between time points T_x_ and T_x+1_ (e.g. weight loss between T_baseline_ and T_12m_) were calculated using the following formula: [(T_x+1_ value – T_x_ value)/T_x_ value].

### Hierarchical clustering to stratify pre- and post-RYGB T2D severity

Groups of T2D severity were built by integrating 7 clinical variables selected based on routine association with T2D severity in clinical settings, which have also been associated with T2D non-resolution^12,62^: HbA1C, fasting blood glucose, T2D duration, patients’ age, and sex, as well as T2D treatments information (total number of glucose-lowering agents and insulin requirements). This integration was performed using Principal Component Analysis (PCA from FactoMineR) at each time point concomitantly, to ensure that clusters’ definitions were constant throughout the follow-up. Three PCA components were kept (Kaiser-Guttman rule, eigenvalues ≥1). Clusters of T2D severity were then defined by using Hierarchical Clustering on Principal Components (HCPC from FactoMineR) and were subsequently named as “Severe” or “Mild” according to clusters’ clinical characteristics, presented in Fig. S3.

For time-sensitive parameters (age, T2D duration), baseline values were used for each time point for the PCA projection to avoid an increase in T2D severity simply due to time passing. The HCPC algorithm initially proposed to divide our cohort into three clusters. However, one of the clusters was gathering all male patients from the cohort, whereas the two others were all female. As such, we decided to reduce the number of clusters to two, among which male patients were homogeneously distributed. Removing sex from the PCA projection and HCPC classification did not induce any changes in our patients’ cluster assignments.

### Human and murine gut microbiome analysis

Details about fecal DNA extraction, library preparation, ONT MinION sequencing, and bioinformatic processing are extensively described in the study by Alili *et al*.^25^

### Fecal DNA extraction

Frozen stool samples were weighted (200 mg of human samples or 20–40 mg of murine samples) and underwent both chemical and physical lyses twice to extract most of the nucleic acids. Samples were deproteinated using Proteinase K (ref 3115844001 – Roche Diagnostics), treated with DNAse-free RNase (ref 12091021 – Invitrogen), and further purified on the PureLink™ Microbiome DNA Purification (ref A29790 – Invitrogen) kit’s columns after steps of precipitation/resolubilization. Fecal DNA purity was assessed using a NanoDrop™ ND-1000 spectrophotometer (ThermoFisher Scientific), its concentration was determined using a Qubit™ fluorometer (Invitrogen™) and fragments integrity using the BioAnalyzer™ (Agilent) with the DNA 12000 kit.

### Bioinformatic data processing – taxonomic binning

Sequence files were used for taxonomic binning using two different reference resources. The first one relies on Centrifuge for the taxonomic binning of individual reads, based on a comprehensive reference database of more than 8,000 reference genomes from prokaryotes and viruses (including both human and mouse reference genomes). To remove spurious taxonomic assignments, we further mapped the reads against their corresponding reference genome using Minimap2 with map-ONT option optimized for Oxford Nanopore reads^63^ to filter spurious assignments in the first step. Only sequences classified by Centrifuge and aligned against the corresponding reference genome with Minimap2 were retained. Species abundance tables were finally generated by summing the counts of each NCBI taxonomic IDs from the minimap2-filtered Centrifuge results. Reference taxonomic tables were reconstructed from Centrifuge NCBI taxonomic IDs using the taxize package into a S4 phyloseq object.

More than 31 million reads were generated for our 188 samples (100 patients from the 5-years cohort, 36 patients from the baseline cohort, and 52 mice from the murine experiment), with an average read size of 2.5 kb. For all human analyses, the number of reads was rarefied with seed to 48,000 reads per sample before analyses, a threshold allowing for the inclusion of all patients while ensuring similar sequencing depth. For the recipient animals, two sets of rarefactions were performed (while ensuring that the seed was the same for each rarefaction): one at 65,000 reads for comparing the donors and their recipient animals, and one at 113,000 reads for within-animal comparisons, to use the maximum sequencing depth possible. Throughout all analyses, the same seed (number 711) was employed to ensure results’ reproducibility of results with random components.

### Bioinformatic data processing – functional analysis

The abundances of KEGG orthology groups (KO) were inferred based on abundances of bacterial species. Briefly, we retrieved the KO content of each KEGG genomes using the KEGG API,^64^ for which species-level pangenomes were reconstructed for all species-level bins based on NCBI taxonomy, and subsequently matched with genomic sequences in the Centrifuge database. Using this matching, the abundance of each KO groups was determined as the sum of the abundances of all species containing the KO of interest. The KO matrix was then collapsed in pathways following KEGG’s different levels of annotations.

### GM data analysis

One patient from the 5-years cohort, specifically from the 5y-Mild cluster, had to be excluded from all analyses, as they were a major outlier (true outlier detected using a rosnerTest (package EnvStats) with k = 5 (5% of the total number of patients) and ɑ = 0.001) on 5/16 of the species differing across T2D severity clusters (Figure 2).

dbRDA were performed using capscale from the vegan package with an inter-genus Bray-Curtis β-diversity matrix as entry, itself computed using vegdist from the same package. Cliff’s delta effect-size estimates were determined using cliff.delta from the effsize package. All analyses on abundance data were performed after rarefaction and exclusion of rare taxa (detected in less than 20% of the samples). Relative abundances values were calculated for each sample by dividing the rarefied abundances by the sum of the filtered abundances.

To assess the implantation of the human GM in recipient animals, we separately rarefied (at 65,000 reads with fixed seed) donors and recipients unrarefied and unfiltered phyloseq objects. Species only present in the donor object were counted, as were species from the recipients’ object. Then, both objects were merged. A presence/absence study was then performed by counting zeros within the merged abundance table. Engraftment of the human microbiota was then determined by dividing the number of common species of the donors and their recipient animals by the total number of species of the human samples.

### Donors’ recall and inoculum preparation for human to mice FMT

Initially, 14 donors were recalled by the medical team of the Nutrition department of the Pitié-Salpêtrière Hospital approximately 5-years after their RYGB. During their clinical visit, fresh stool samples were collected. No patient declared any intake of antibiotics within three months before the feces collection.

Upon propulsion, stool samples were maintained in tightly closed plastic pots under anaerobic conditions (GenBag-anaer™, BioMérieux). Feces were then sampled, weighted, and diluted (10% weight/weight dilution) in an anoxic cryo-preserving solution consisting of 10% glycerol (ref G5516 – Sigma-Aldrich), 10% skim milk (ref 70166 – Sigma-Aldrich), and a cocktail of reducing agents (L-cysteine (1 g/L – C7477), sodium ascorbate (1 g/L – A7631), L-glutathione (0.1 g/L – G4251), and uric acid (0.2 g/L – U2625) – all from Sigma-Aldrich) all diluted in sterile water. This solution was used to promote bacterial survival upon freezing and thawing. Samples were then homogenized using a hand-held Ultra-Turrax homogenizer (T10 Basic Ultra-Turrax™, IKA). Slurries were then filtered on three layers of sterile gauzes and were aliquoted in 5 ml tubes before being stored at −80°C until usage.

### Mouse study: experimental design

Male specific-pathogen-free weaning (3 weeks) C57Bl6/j mice were obtained from Charles River Laboratories (France) and were homogeneously distributed in cages according to their body weight, with 4 animals per cage (one cage per human donor). Animals were maintained on a 12-hour light-dark cycle with ad-libitum access to water and a “western” diet enriched in lipids and sucrose (19.5% w/w protein (mostly casein), 35% w/w sucrose, 5% w/w fibers, 21% w/w fat (mostly butter) – D12079Bi from Research Diet). Mice were housed in individually ventilated cages, which were changed every week. Food, bedding, and enrichments were irradiated at 10 kGy. Drinking water was filtered and treated with 2 ppm chlorine. Mice were followed for 14 weeks before sacrifice and tissue harvesting. Cages were systematically handled in the same order and under a dedicated hood with a laminar flow; gloves were changed between the handling of each cage, and all instruments and working area were disinfected with hydrogen peroxide 3% vol/vol to prevent cross-contaminations.

Importantly, one cage of animals (corresponding to the 14th donor) had to be excluded from the study due to a leak in its water supply. These 4 animals had to be euthanized due to drastic and rapid weight loss and were thus excluded from all analyses presented in this paper. A total of 52 animals were therefore studied, 16 being recipients from 5y-Mild donors and 36 being recipients from 5y-Severe donors (Table 2).

### Study approval

All procedures were validated by the Comité d’Éthique pour l’Expérimentation Animale n°5 under agreement number #25963.

### Body weight and composition measurements and fecal pellets harvesting

Body weight and food intake were monitored once a week using the same balance (Scout SPX, OHAUS). Body composition was assessed every two weeks using a LF90 Minispec+ scanner (Brucker). Stool pellets were collected every two weeks and were immediately placed in liquid nitrogen before being stored at −80°C.

### Antibiotics-induced gut microbiota depletion and fecal matter transfers

To deplete the endogenous GM of our animals before the inoculation, all animals were gavaged with a cocktail of broad-spectrum antibiotics over 7 days^26^ upon their arrival at the animal facility. As we want the inoculation to happen as close as possible to the weaning period, mice did not have any acclimatization before the beginning of this procedure.

The antibiotic solution containing 200 mg/kg of ampicillin (ref A9393 – Sigma-Aldrich), neomycin (ref N1876 – Sigma-Aldrich), and metronidazole (ref M1547 – Sigma-Aldrich), as well as 100 mg/kg of vancomycin (ref V2002 – Sigma-Aldrich) diluted in sterile tap water was prepared and stored at −20°C until usage. Antibiotics were administered once a day in the morning for a week. During that time, cages, bedding, and food pellets were renewed regularly (every day for the first three days, then every two days) to prevent re-contaminations, as mice are coprophagic. In the afternoon of the 7th day, animals received 700 µL of a solution of polyethylene glycol (PEG), a laxative treatment used to flush out the residual microbiota and antibiotics from the intestinal lumen. The PEG solution was prepared using Colopeg™ (Bayer), which contains PEG 3350 (77.5 g/L), sodium chloride (1.9 g/L), sodium sulfate (7.4 g/L), potassium chloride (0.98 g/L), and sodium bicarbonate (2.2 g/L). The laxative powder was diluted in sterile tap water (according to the manufacturer’s instructions), filtered on a 0.22 µm membrane, and stored at −20°C until usage. The administration was divided in two equal volumes that were gavaged within 1 hour after a 2-hours fast. The PEG treatment was performed a second time in the morning of the 8th day following the same procedure. Throughout the microbiota depletion protocol, animals were closely monitored and showed no sign of suffering.

For the inoculations, frozen tubes (1 per donor per day) were thawed in ~30°C water for 30 seconds and were agitated once by inverting before opening. 300 µL of fecal slurries were then inoculated to each animal by gavage for three consecutive days. To ensure long-lasting microbiota engraftment, one gavage with FMT solutions was performed every 4–5 weeks during the follow-up (see Figure 3a).

### Mice metabolic phenotype exploration

An oral glucose tolerance (OGTT) was performed 11 weeks after the initial inoculation. Mice were fasted for 6 hours in clean cages with *ad-libitum* access to water but not food. Once fasted, all animals were gavaged with 2 g/kg of a 30% glucose solution (Lavoisier). Blood glucose levels were measured using Accu-Chek® PERFORMA glucometer (Roche Diabetes Care) through tail clipping 30 minutes before and at the moment of the gavage, as well as 15, 30, 60, and 90 minutes after. Furthermore, 30 µL of whole blood were drawn using EDTA-coated capillaries (16.444 – Sarstedt) at baseline, 15, 30, and 60 minutes after the gavage for further cytokine quantification. Plasma samples were collected by centrifuging blood samples at 13,000 g for 3 minutes at 4°C and were kept at −20°C until usage. At the end of the experiment, animals were placed back into their respective cages with *ad-libitum* access to both food and water. The area under the curve (AUC) of the OGTT was determined using the trapezoidal method. A constant area value was subtracted to all AUCs, representing the area below the smallest blood glucose value. HOMA-IR and QUICKI insulin resistance/sensitivity indexes were computed using the following formulas: [HOMA-IR = (fasting blood glucose (mg/dl) * 0.0555 * fasting insulin (mU/L))/22.5]; [QUICKI = 1/(log(fasting blood glucose (mg/dl)) + log(fasting insulin (mU/L))].

### Intestinal energy absorption

Thirteen weeks after the inoculation, mice were placed on metal grids within their cages emptied of any beddings. Feces and food residues that passed through the grids were collected and weighed every 24 hours for 3 consecutive days. During the experiment, both food intake and spillage were monitored daily. Feces were dried at 37°C for 48 hours and re-weighed. Total caloric content (kcal/g) of both the diet and feces was determined by bomb calorimetry (C200™, IKA) at the UMR1388 GenPhyse (INRAE Occitanie Toulouse, France). The net intestinal energy absorption capacity is expressed as a percentage of total energy ingested and represents the proportion of ingested energy that was not recovered in feces.

### Insulin quantification

Circulating insulin levels were quantified in plasma samples collected during the OGTT using a MILLIPLEX™ Mouse Metabolic Hormone Panel – Metabolism Multiplex Assay (Merck) using a Luminex™ (LX200) following manufacturer’s instructions.

### Statistics

All analyses were conducted using Rstudio 3.10 relying on R 3.6.2. P-values were corrected to control for multiple testing errors using the Benjamini-Hochberg method. P or q-values were considered significant when ≤ 0.05 (or ≤ 0.1 when clearly stated).

### Human clinical study

Continuous variables are expressed as mean ± standard deviation and categorical variables as numbers (percentages) unless stated otherwise. Comparisons of continuous variables were performed by ANOVA with the effect of sex and age accounted for. For categorical data comparisons, binomial models controlling for sex and age were used. Missing data were imputed by the variable’s median.

### Human to mice fecal microbiota transfer experiment

To avoid statistical imprecision due to pseudoreplication (artificial inflation of the number of samples, as multiple animals are recipients of the same donor), all statistical analyses on data concerning the recipient animals were performed using the geometric mean of each donor’s 4 animals (unless specified otherwise). As such, 13 mean samples (corresponding to the 13 donors) were used for comparisons. Continuous variables are presented as the mean ± error of the mean unless specified otherwise. Parametric tests (Welch Two-Samples T-tests) were used solely on normally distributed data with homogeneous variance. When assumptions were not met, non-parametric tests were employed (Wilcoxon/Kruskall-Wallis rank-sum tests).

## Supplementary Material

Supplemental Material

## Data Availability

The raw data of libraries generated during this study are publicly available at the European Nucleotide Archive (ENA) under accession number PRJEB49428 (https://www.ebi.ac.uk/ena/browser/view/PRJEB49428). Detailed sample metadata are available in Supplementary Tables S5 and S6. More detailed data are available upon reasonable request.
