# Supplementary material for: The human gut microbiota contributes to type-2 diabetes non-resolution 5-years after Roux-en-Y gastric bypass
Source: Gut Microbes. 2022 Apr 18;14(1):2050635. doi: 10.1080/19490976.2022.2050635 (PMC9037437; doi:10.1080/19490976.2022.2050635)
Supplement: Supplemental Material [file KGMI_A_2050635_SM0397.zip › Revised supplementary materials Debedat et al 20212.docx]

## Supplementary figures and tables


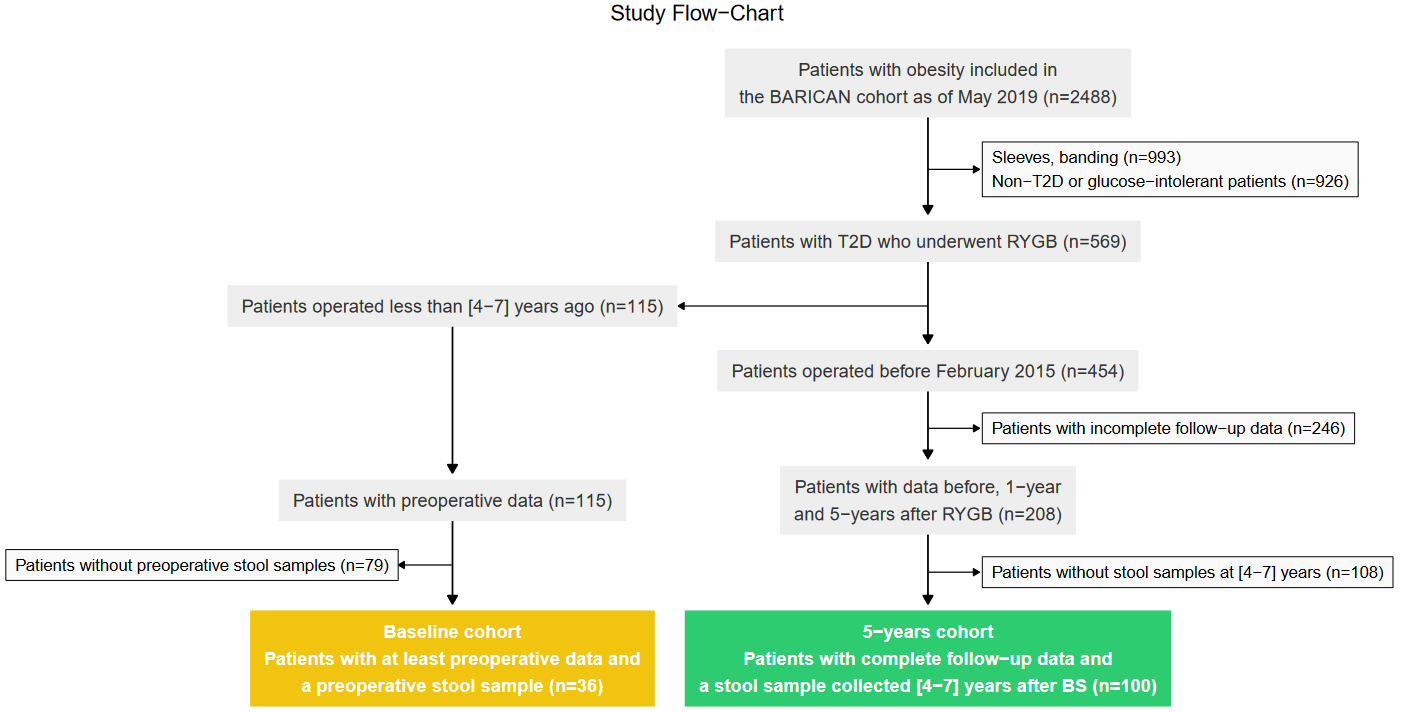


###### Fig. S1. Study flow-chart. BS, bariatric surgery ; RYGB, Roux-en-Y gastric bypass; T2D, type-2 diabetes.


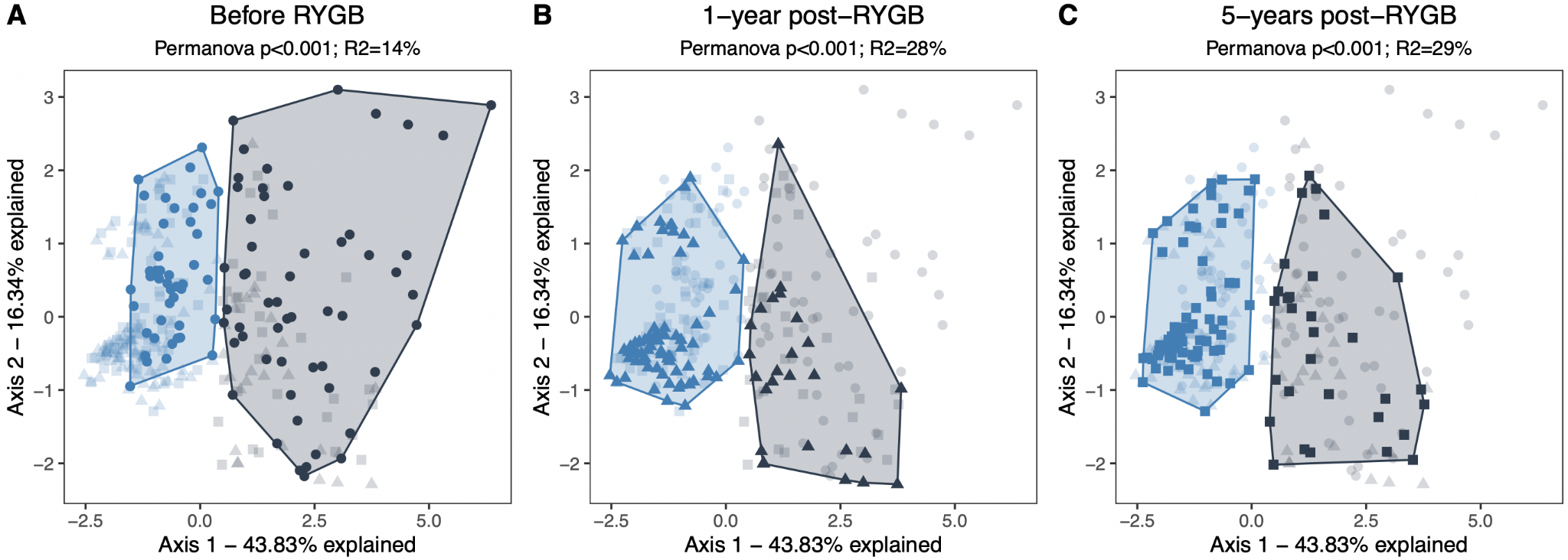


###### Fig. S2. Time-specific PCA representations of T2D severity clusters. Patients’ positions according to their T2D severity (A) before RYGB, (B) at 1-year after, and finally (C) 5-years after the surgery. RYGB, Roux-en-Y gastric bypass; T2D, type-2 diabetes.


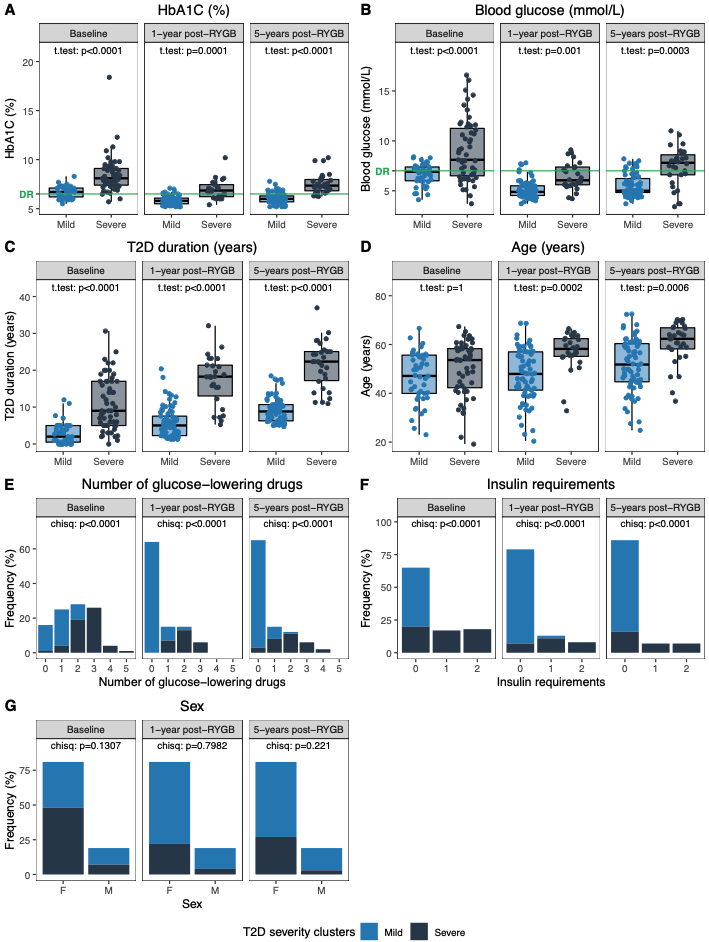

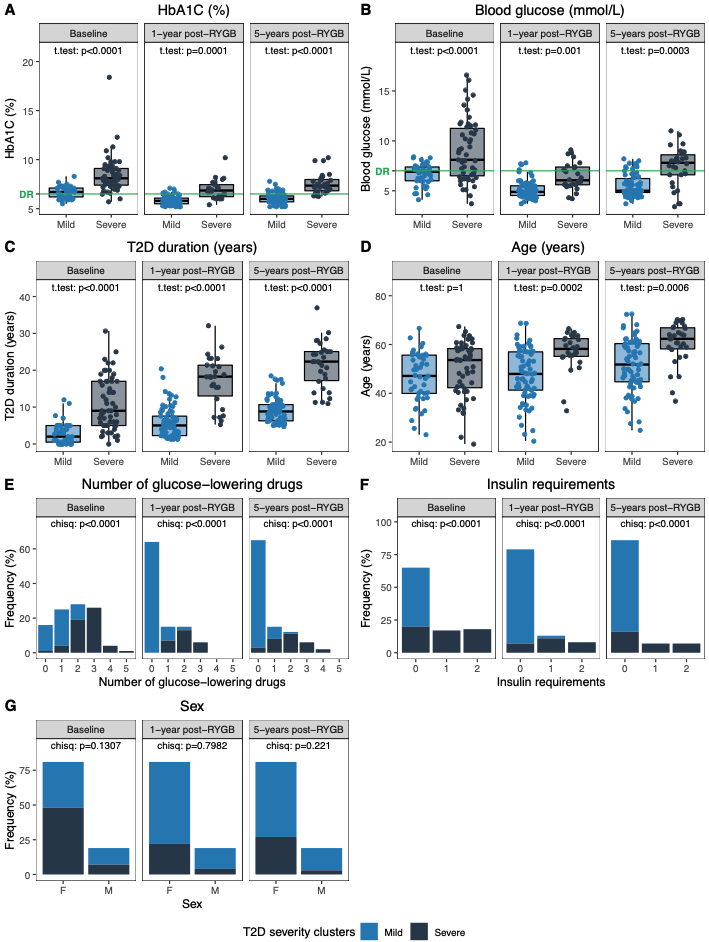


###### Fig. S3. Differences of variables used to construct T2D severity clusters at each time-point of follow-up. (A) HbA1C levels (%). (B) Fasting blood glucose levels (mmol/L). (C) T2D duration (years). (D) Patient’s age (years). (E) Number of total glucose-lowering drugs (n). (F) Insulin requirements (n). (G) Patient’s sex. For (A) and (B), the green line indicated T2D remission thresholds as proposed by Buse *et al*. DR, type-2 diabetes remission; F, female; M, male; RYGB, Roux-en-Y gastric bypass; T2D, type-2 diabetes.


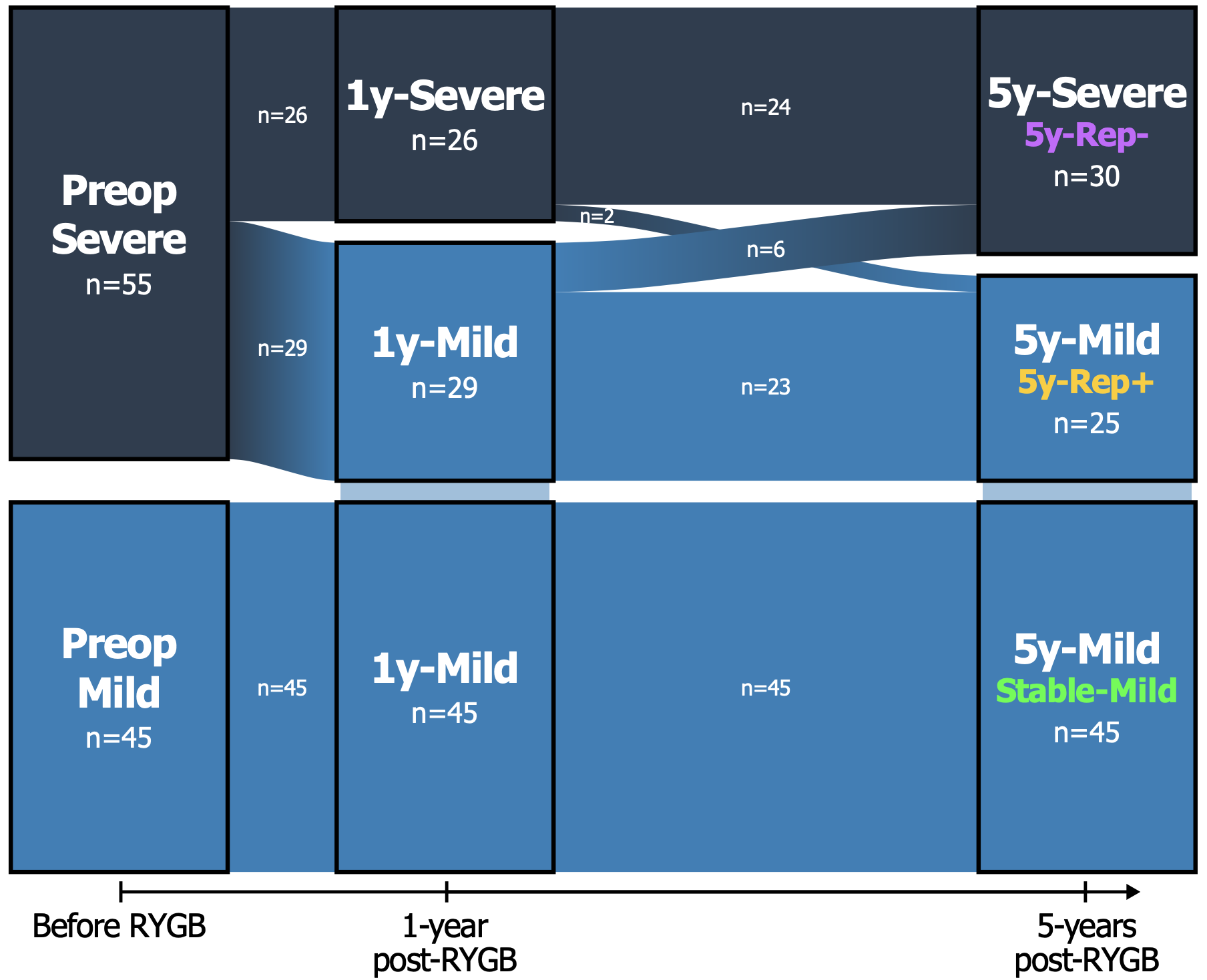


###### Fig. S4. Changes of T2D severity in RYGB patients during the follow-up. Numbers written on alluviums indicate the number of patients following this path. 1y, 1-year; 5y, 5-years; Rep+, good responders (i.e. Severe at baseline and Mild at 5-years); Rep-, poor responders (i.e. Severe at all times); RYGB, Roux-en-Y gastric bypass.


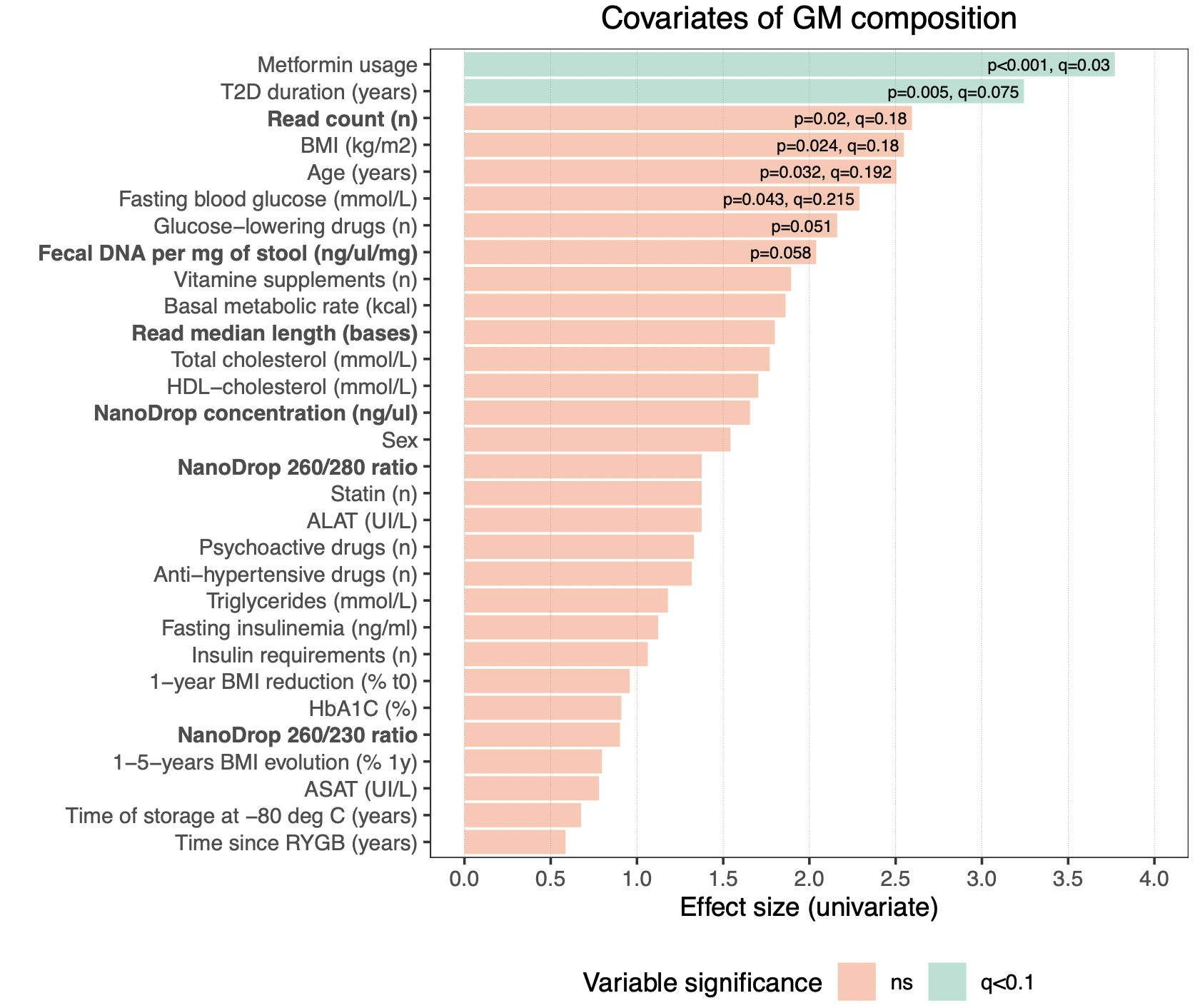


###### Fig. S5. Clinical and technical covariates of GM composition in the human cohort. Effect-size of GM variation explained in univariate models of distance-based Redundancy Analyses (dbRDA) based on a genus-level ß-diversity matrix. Bolded variables are associated with the methods used to sequence fecal DNA (fecal DNA extraction, sequencing efficiency). ALAT, alanine aminotransferase; ASAT, aspartate aminotransferase; BMI, body mass index; GM, gut microbiome; HDL, high-density lipoprotein; RYGB, Roux-en-Y gastric bypass; T2D, type-2 diabetes.

######
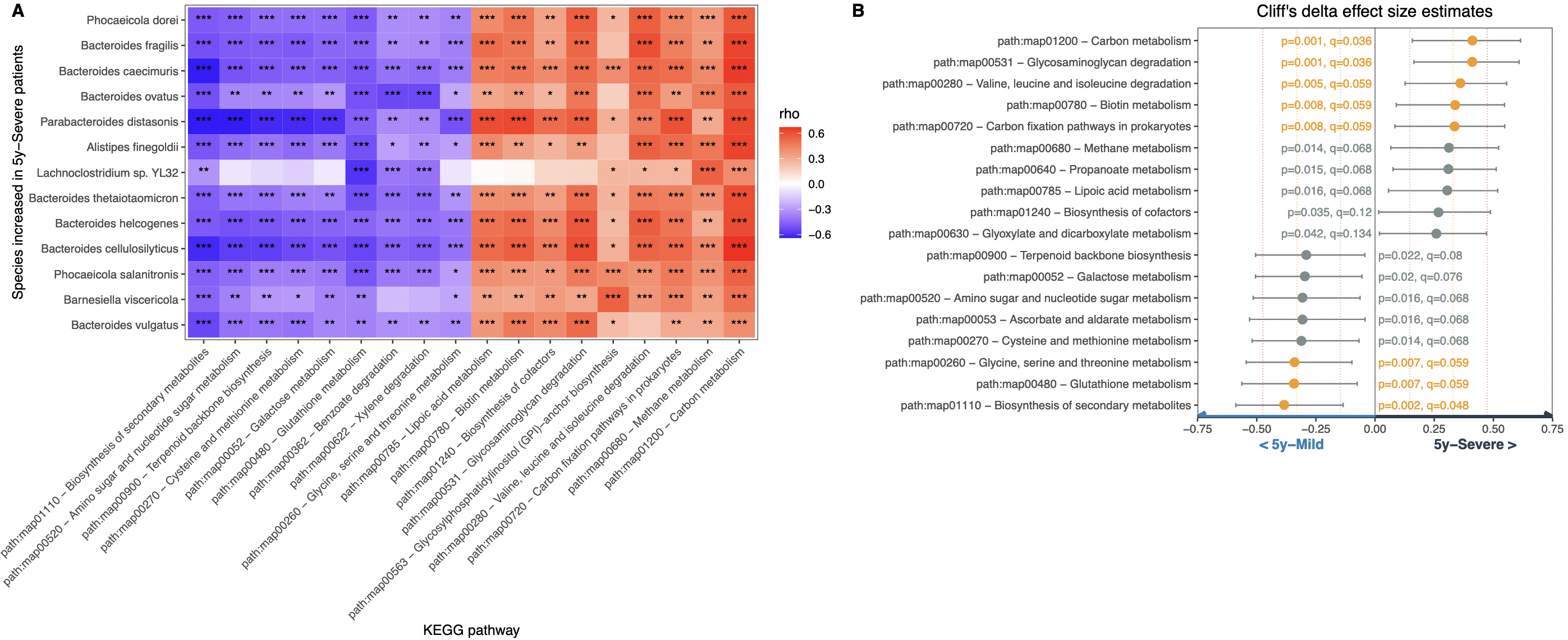


###### Fig. S6. Functional differences within the gut microbiome of patients across T2D severity clusters. (A) Spearman rank correlations between the abundances of KEGG-pathways and bacterial species increased in 5y-Severe patients. Only correlations with at least one FDR-adjusted pvalue <0.05 and with Rho below -0.5 or greater than 0.5 were included in the figure. (B) Cliff’s delta effect size estimates, 95% confidence intervals and Welch Two-Samples t-tests p-values across T2D severity clusters and inferred metabolic pathways. ***: q<0.001; **: q<0.01; *: q<0.05


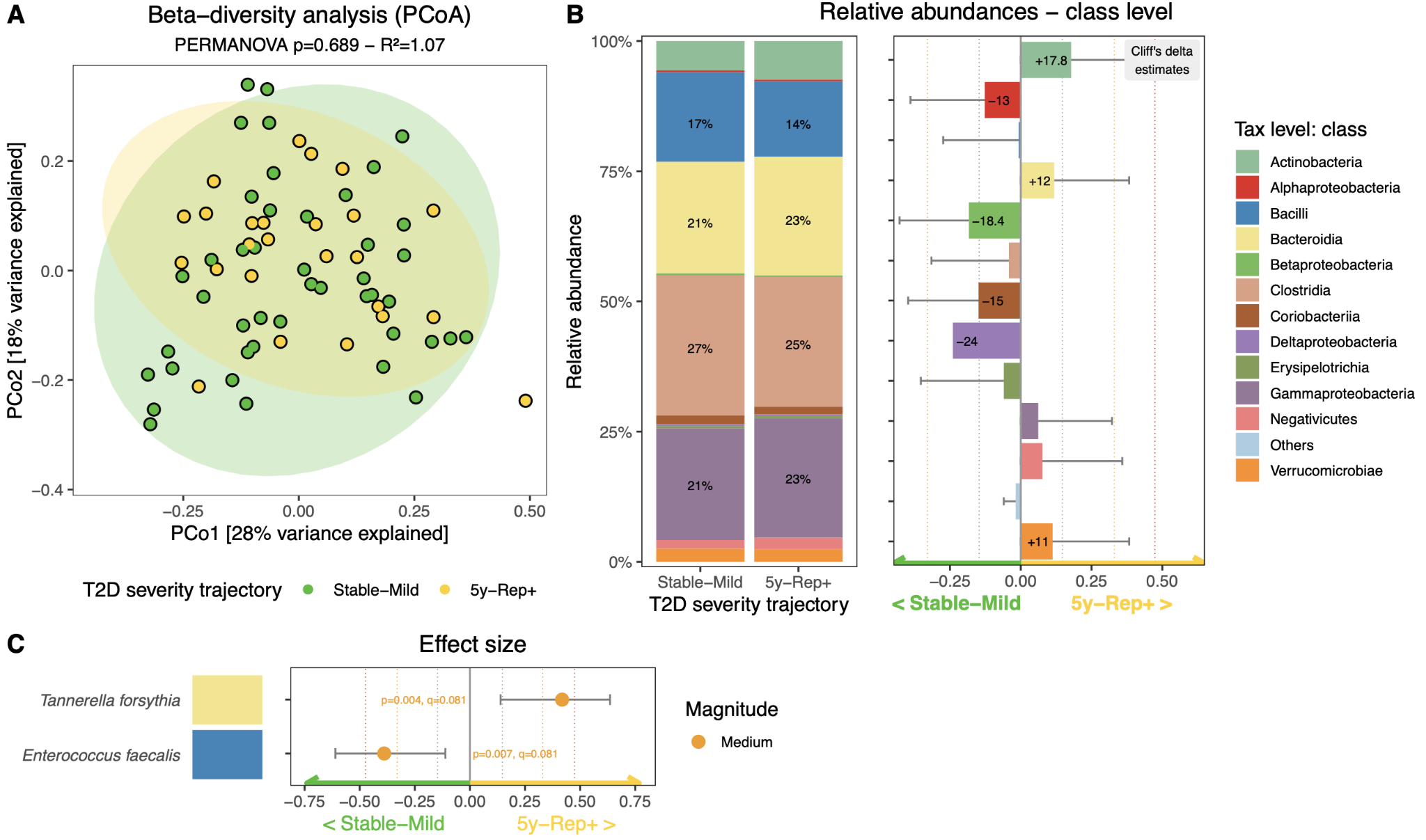


###### Fig. S7. Minor GM differences are observed according to 5y-Mild patients’ trajectory of metabolic improvements. (A) Genus-level community ordination (PCoA based on Bray-Curtis β-diversity matrix). (B) Left: Classes’ abundances of Stable-Mild and 5y-Rep+ patients (n=44 and 25, respectively). Right: Cliff’s delta effect size estimates and 95% confidence intervals between Stable-Mild and 5y-Rep+ patients (n=44 and 25, respectively) across classes representing at least 0.1% of the total ecosystem (n=13). (C) Plot of significant (p<0.05, q<0.1) and non-negligible Cliff’s delta effect sizes between Stable-Mild and 5y-Rep+ patients (n=44 and 25, respectively) across bacterial species representing at least 0.1% of the total ecosystem (n=95). Rectangles next to the taxonomic names represent species’ classes as in Figure S7B. 5y, 5-years; PCoA, Principal coordinates analysis; T2D, type-2 diabetes.


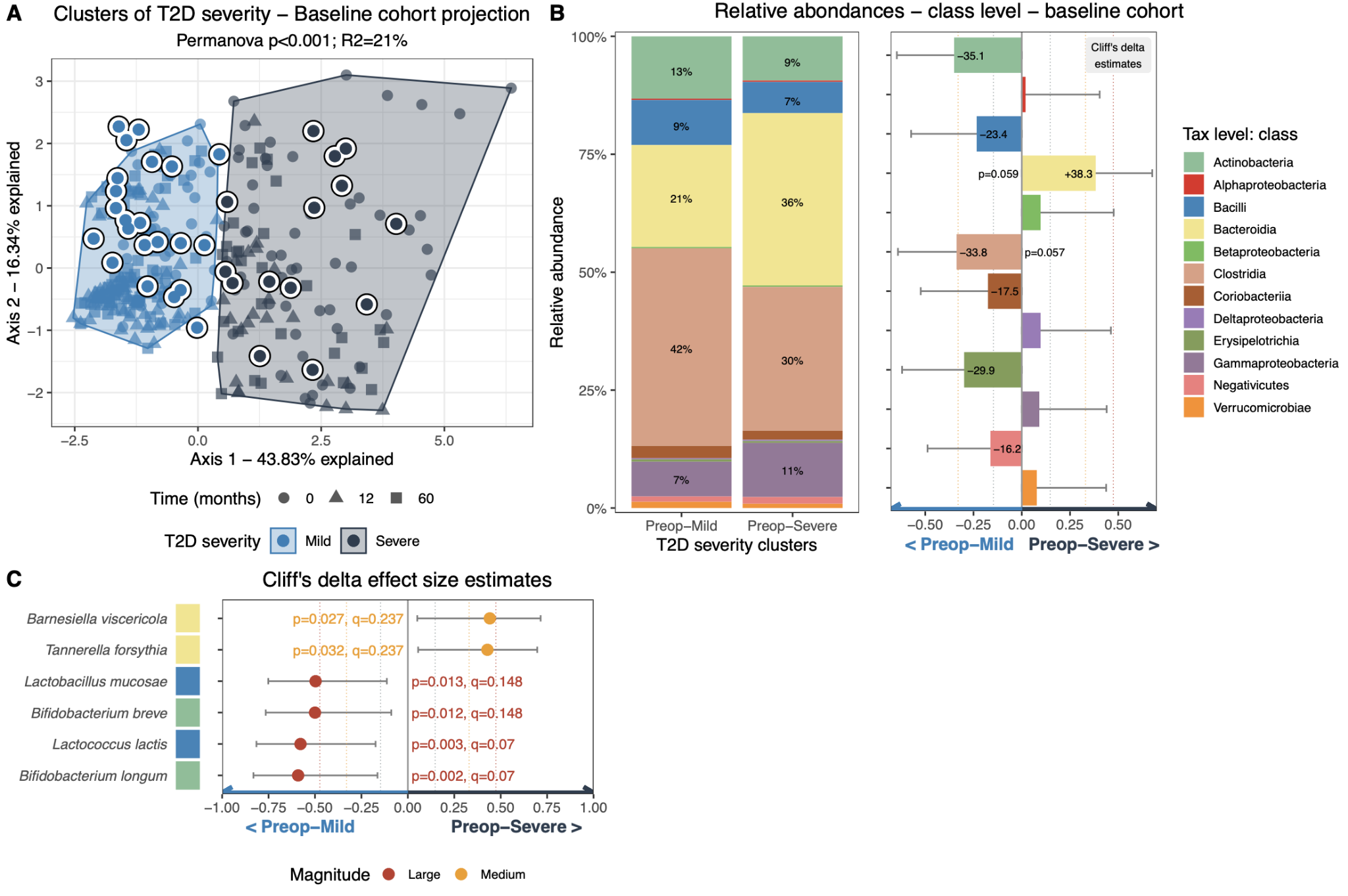


######

###### Fig. S8. Baseline GM composition of n=36 patients from the baseline independent cohort according to their T2D severity. (A) PCA representations of T2D severity clusters of our main cohort (Figure 1A), including the 36 patients (highlighted in circles with white borders) from the baseline cohort projected onto the original PCA. Patients from the baseline cohort were assigned to either Preop-Severe or Preop-Mild according to their relative position on the PCA of the main cohort. (B) Left: Taxonomic classes’ relative abundances of patients across baseline T2D severity clusters. Cliff’s delta effect size estimates and 95% confidence intervals between Preop-Mild and Preop-Severe patients of the baseline cohort (n=22 and 14, respectively) across classes representing at least 0.1% of the total ecosystem. (C) Cliff’s delta effect size estimates and 95% confidence intervals across T2D severity clusters and bacterial species representing at least 0.1% of the total ecosystem (n=75). Rectangles next to the taxonomic names represent species’ classes, colored according to Figure S8B. 5y, 5-years.


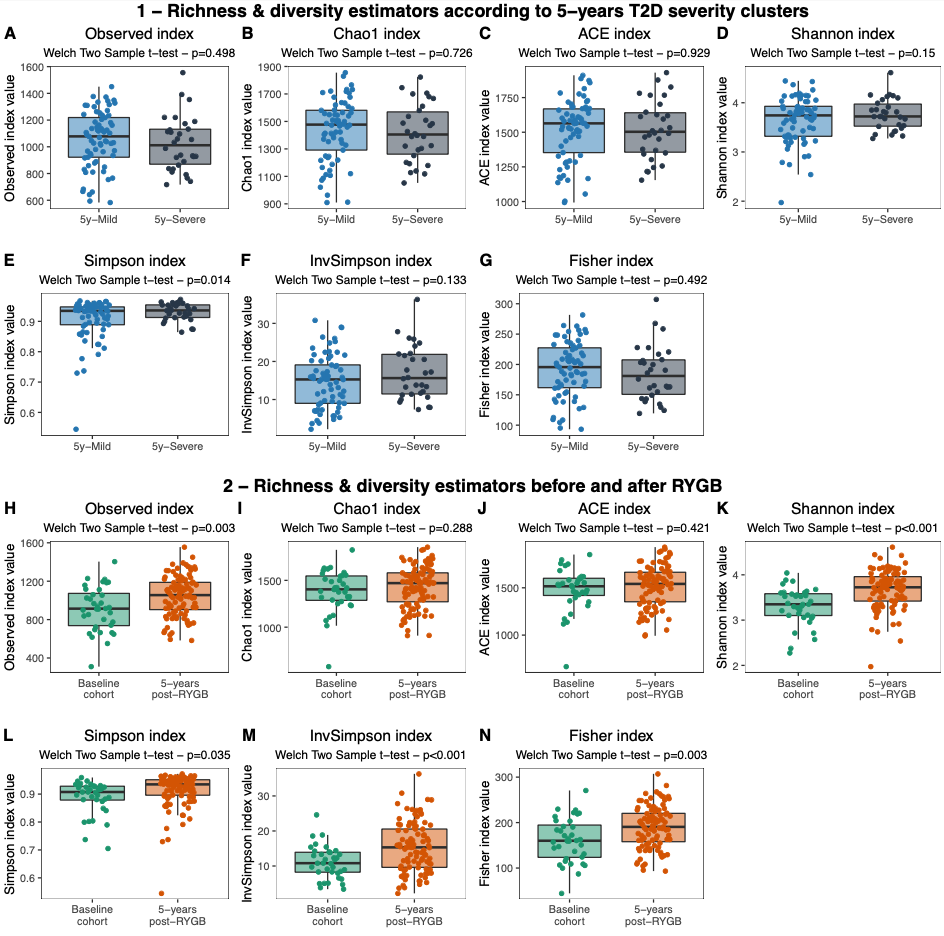


######

###### Fig. S9. No differences in GM diversity or richness can be observed across T2D severity, despite post-RYGB increases for both parameters. (A-G) Comparison of GM richness and diversity indexes across 5-years T2D severity clusters. (H-N) Comparison of GM richness and diversity indexes between our cohort of patients 5-years after their RYGB (n=99), and the baseline cohort, which gathers n=36 patients. 5y, 5-years; ACE, abundance-based coverage estimator; RYGB, Roux-en-Y gastric bypass; T2D, type-2 diabetes.


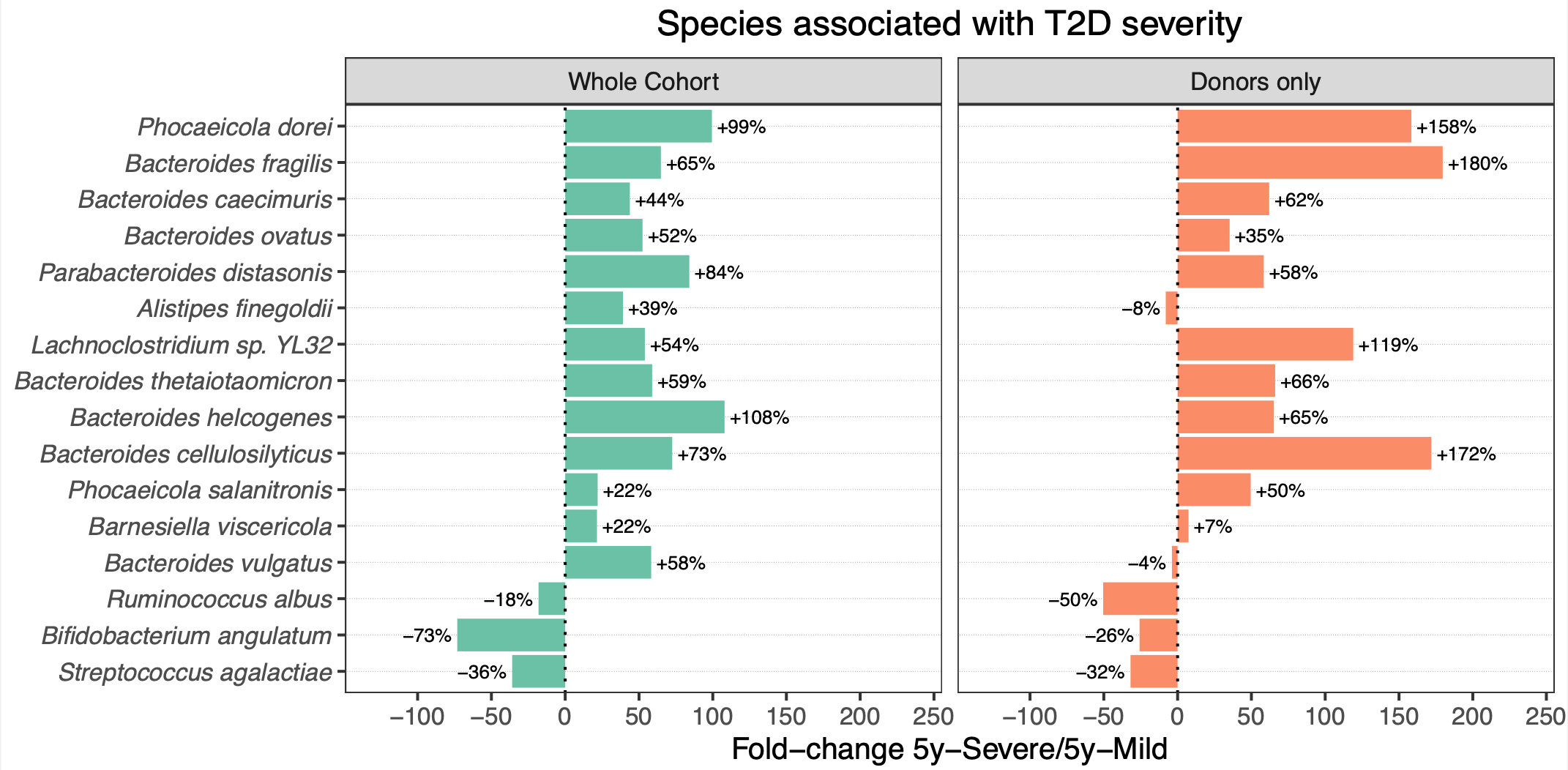


###### Fig. S10. Abundances of bacterial species associated with T2D-severity in the selected donors are representative of those of the whole cohort. Fold-changes were computed as follows: [(mean(Abundance 5y-Severe) - mean(Abundance 5y-Mild))/(mean(Abundance 5y-Mild))]. Only species from Figure 2D are represented. 5y, 5-years; T2D, type-2 diabetes.


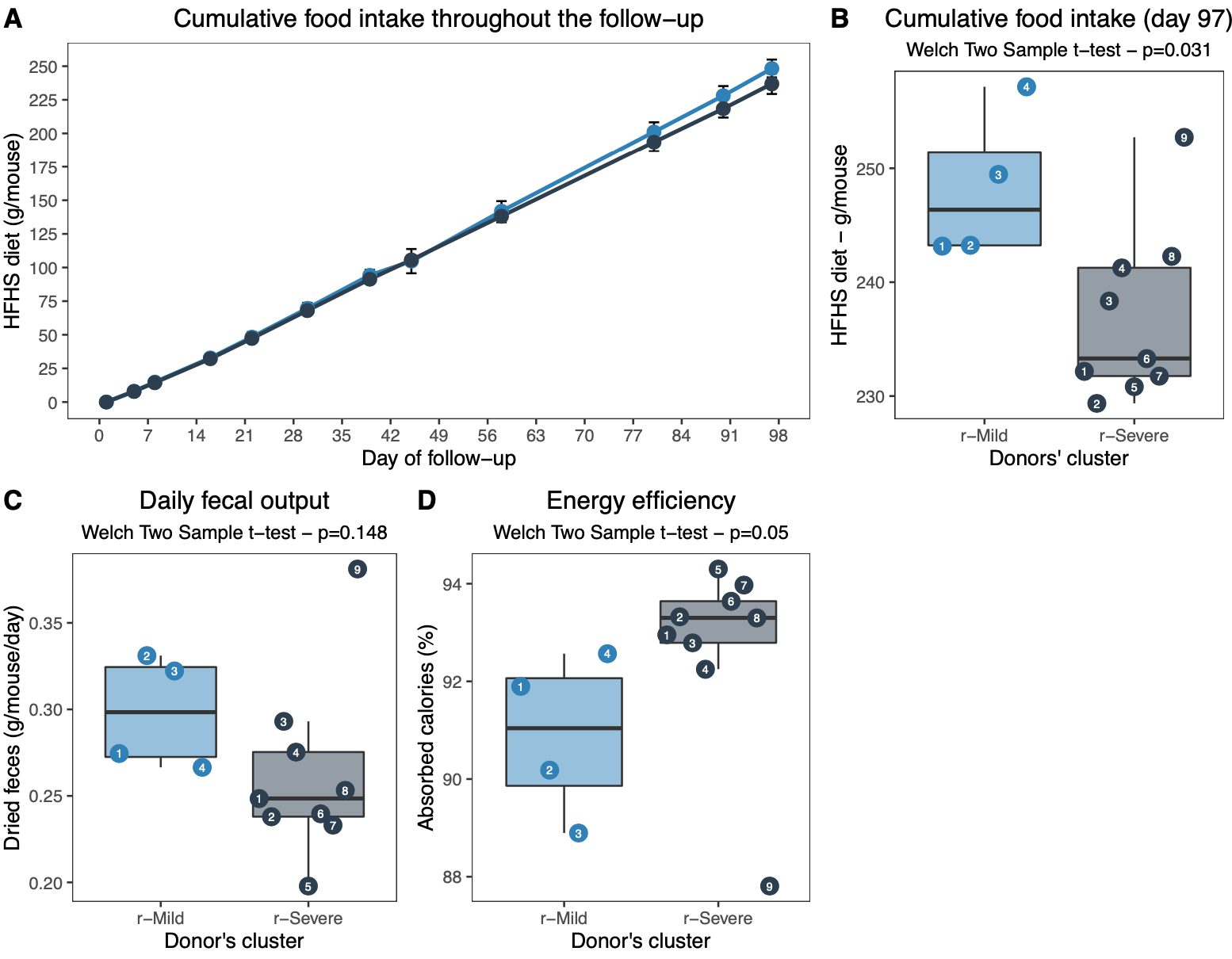


######

###### Fig. S11. Differences in food intake of recipient animals are marginal, whereas caloric absorption and caloric absorption efficiency are decreased in r-Mild animals. (A) Cumulative food intake, determined as the total amount of food eaten throughout the follow-up, divided by the number of animals in the cage. (B) Cumulative food intake at the end of the experiment. (C) Quantity of dried feces produced daily, and measured over 3 days, divided by the number of animals in the cage. (D) Proportion of the total amount of caloric eaten that is not excreted in the feces. HFHS, high-fat high sucrose diet; r-, recipient.


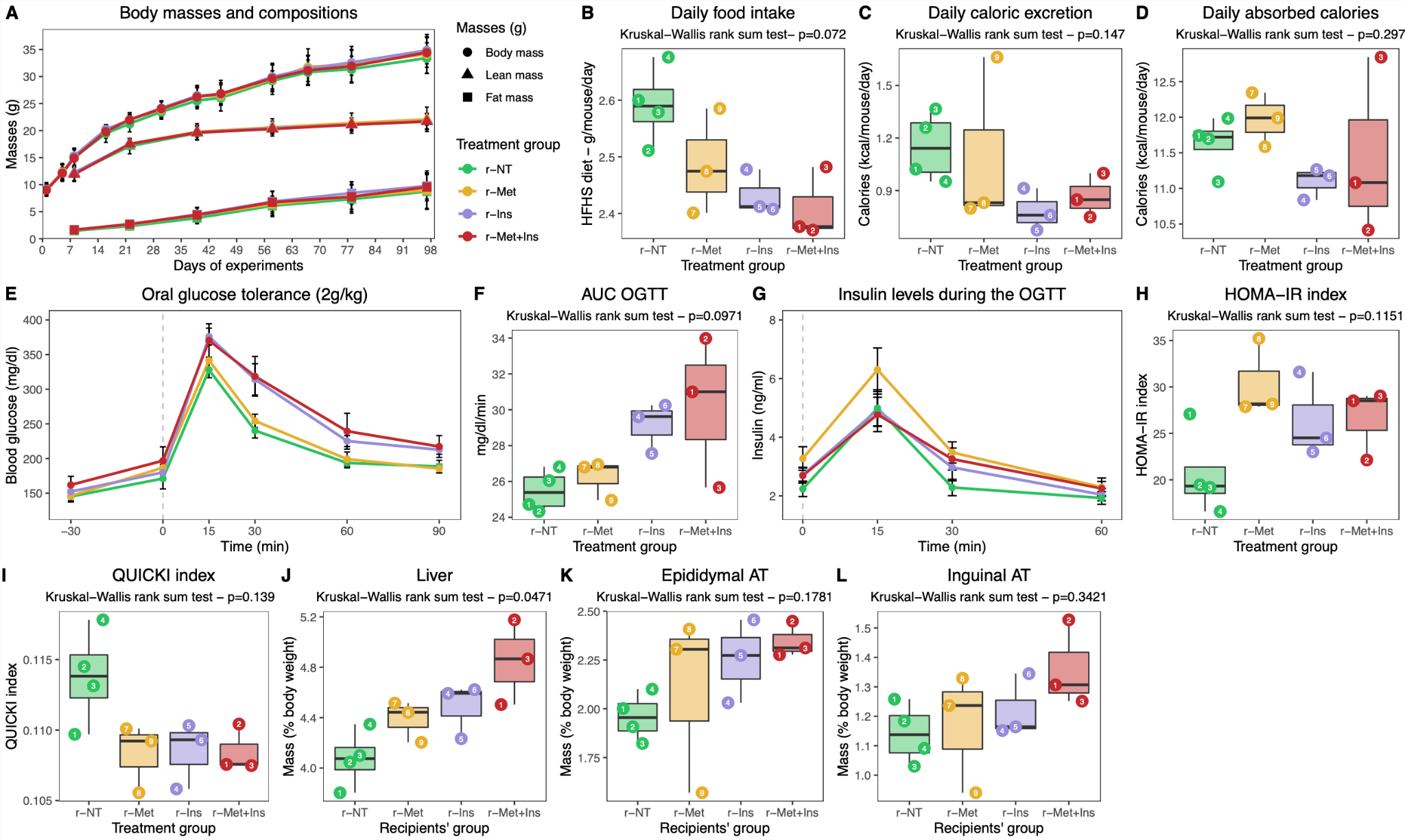


######

###### Fig. S12. Metabolic alterations are variable but mostly independent of donors’ treatments for T2D. (A) Body (rounds), fat (squares), and lean (triangles) mass evolutions throughout the follow-up. (B) Daily food intake. (C) Daily fecal excretion of calories. (D) Daily caloric absorption. (E) Oral glucose tolerance test (dose of glucose: 2g/kg) performed at 11 weeks after the inoculation. (F) AUC of the OGTT. (G) Insulin levels quantified in plasma collected during the OGTT. (H) HOMA-IR index. (I) QUICKI index. (J-L) Liver, epididymal, and inguinal adipose tissues masses. Numbers within the dots on boxplots represent the donor number, as presented in Table 2. AT, adipose tissue; AUC, area under the curve; FMT, fecal microbiota transfer; Ins, insulin; Met, metformin; NT, non-treated; OGTT, oral glucose tolerance test; PEG, polyethylene glycol; r-, recipient.

##
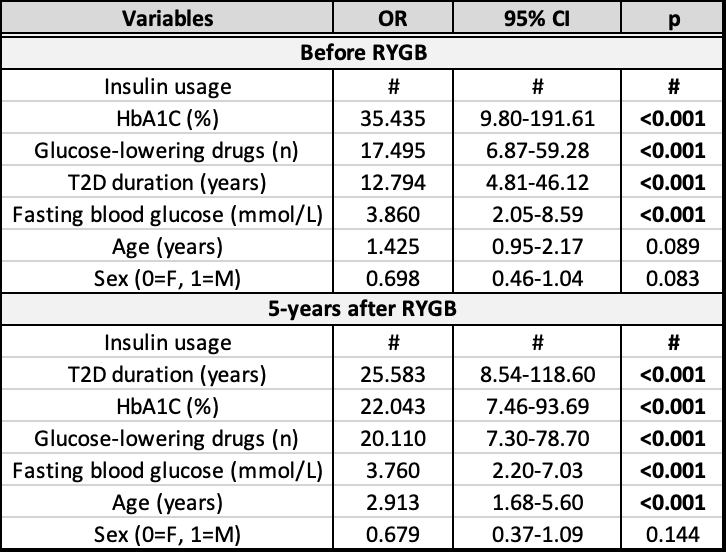


###### Table S1. Standardized odd-ratios evaluating the association with clinical variables and T2D severity. The Mild group was used as reference; these OR thus evaluate the risk of being in the Severe cluster. The variables were standardized using the following formula: z_i_ = (x_i_ - µ)/σ where µ represents the variable’s mean and σ its standard deviation. # These OR could not be computed due to a perfect separation (i.e. all insulin-requiring patients were in the Severe cluster). CI, confidence-intervals; OR, odd-ratios; RYGB, Roux-en-Y gastric bypass; T2D, type-2 diabetes.


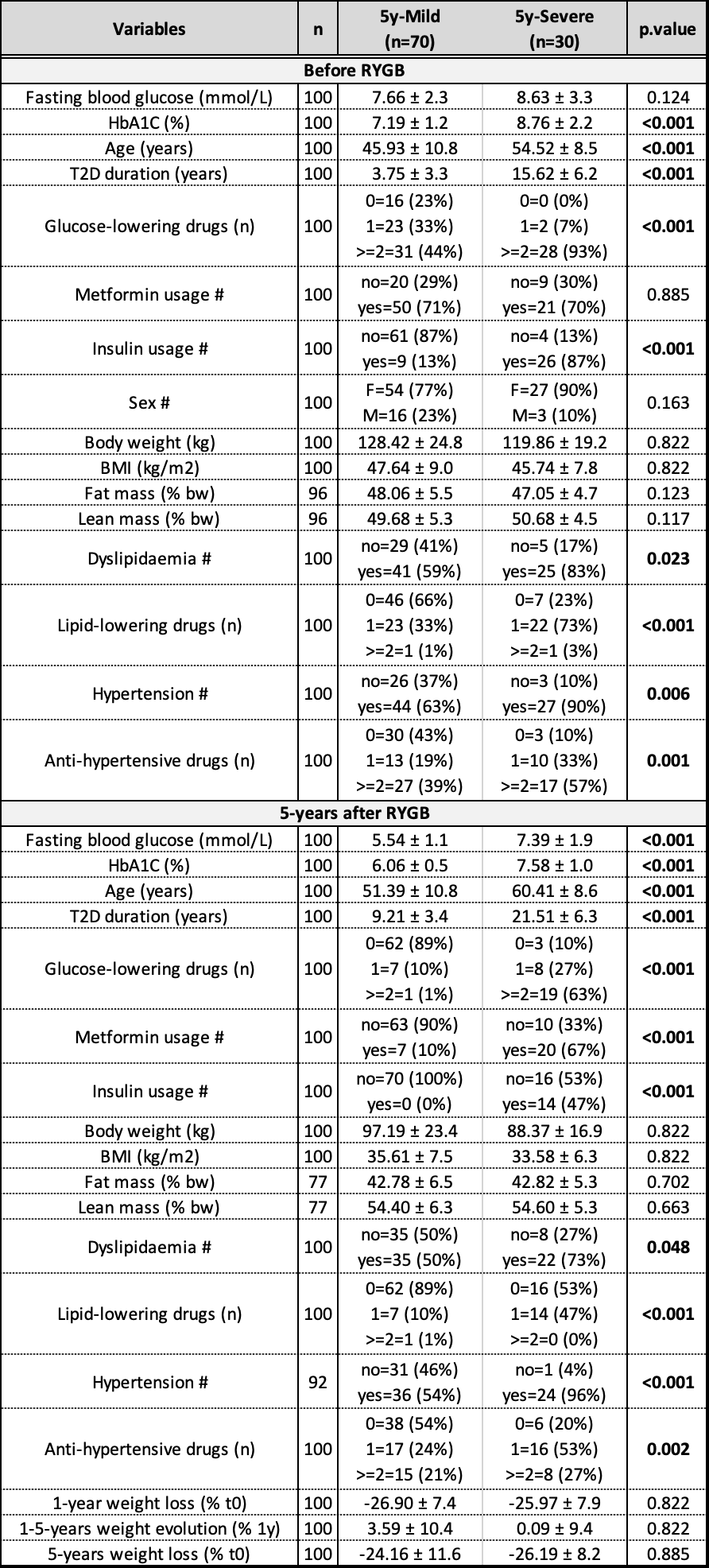


**Table S2. Patients’ clinical chara-cteristics before and after RYGB according to their 5-years T2D severity clusters.**

Values are presented as mean ± standard deviation for continuous variables, or within-classes [n (%)] for categorical variables. For continuous data, ANOVA controlled for age and sex were used to assess overall T2D severity clusters differences. For categorical variables (noted #), binomial models adjusted for age and sex were used to assess differences across classes. P.values were further corrected to avoid multiple comparisons errors using the Benjamini-Hochberg method. BMI, body mass index; bw, body-weight; RYGB, Roux-en-Y gastric bypass; T2D, type-2 diabetes.


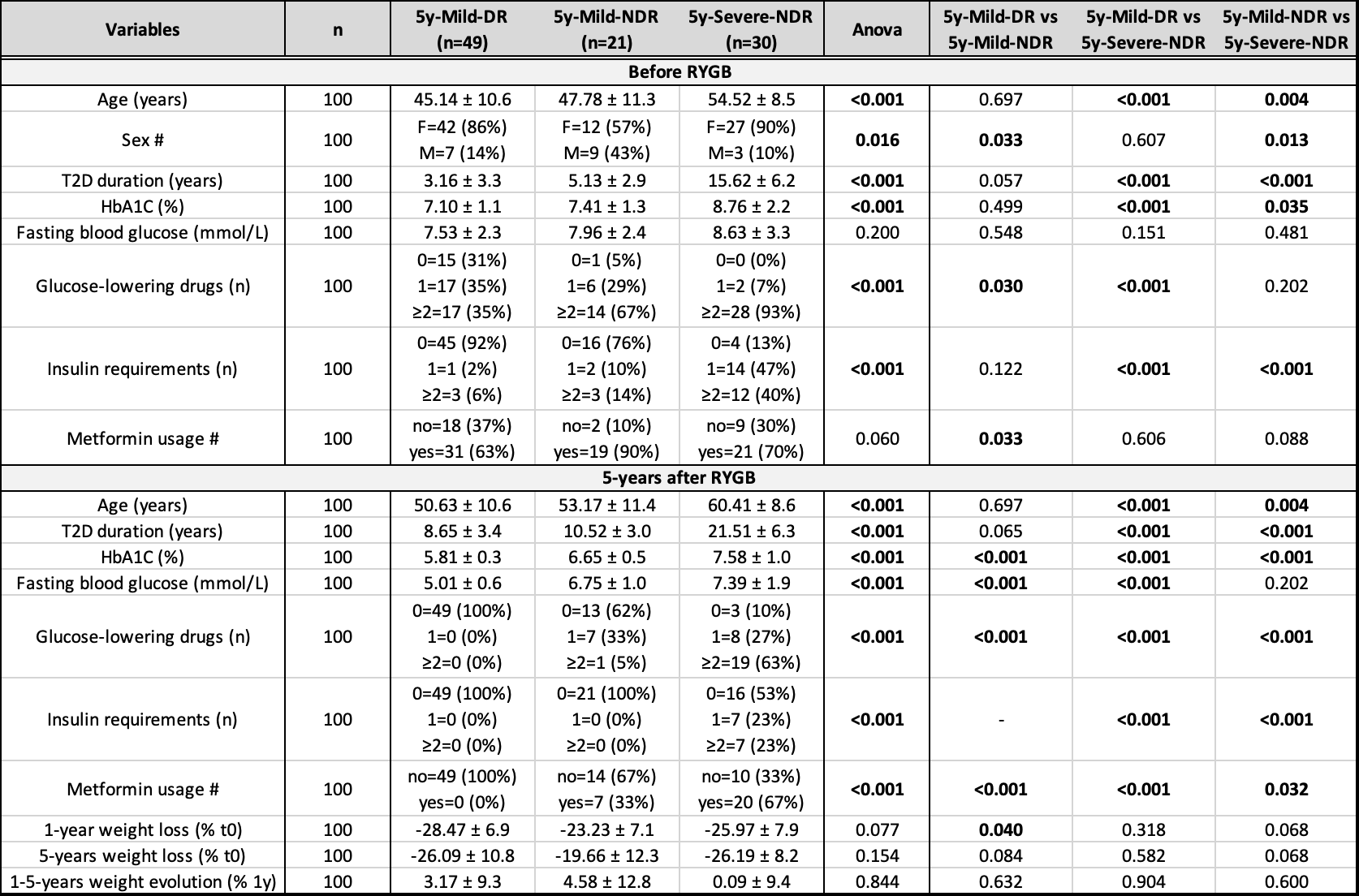


###### Table S3. 5-years T2D severity clusters only differed from DR for n=21 patients. Values are presented as mean ± standard deviation for continuous variables, or within-classes [n (%)] for categorical variables. For continuous data, ANOVA controlled for age and sex were used to assess overall T2D severity trajectories differences, and Tukey tests were used to assess within-trajectories differences. For categorical variables (noted #), binomial models adjusted for age and sex were used to assess differences across classes. P.values were further corrected to avoid multiple comparisons errors using the Benjamini-Hochberg method. 1y, 1-year; 5y, 5-years; BMI, body mass index; DR, type-2 diabetes remission; NDR, non-type-2 diabetes remission; RYGB, Roux-en-Y gastric bypass; T2D, type-2 diabetes.


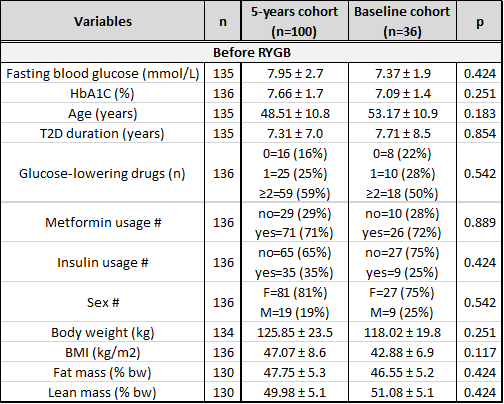


###### Table S4. Comparisons of the pre-operative clinical characteristics of both the 5-years (n=100) and baseline cohorts (n=36). Values are presented as mean ± standard deviation for continuous variables, or within-classes [n (%)] for categorical variables. For continuous data, ANOVA were used to assess cohort differences. For categorical variables (noted #), Chi-2 tests were used to assess differences across classes. P.values were further corrected to avoid multiple comparisons errors using the Benjamini-Hochberg method. BMI, body mass index; bw, body-weight; RYGB, Roux-en-Y gastric bypass; T2D, type-2 diabetes.
